# Coping With Stigma in the Workplace: Understanding the Role of Threat Regulation, Supportive Factors, and Potential Hidden Costs

**DOI:** 10.3389/fpsyg.2019.01879

**Published:** 2019-08-27

**Authors:** Colette Van Laar, Loes Meeussen, Jenny Veldman, Sanne Van Grootel, Naomi Sterk, Catho Jacobs

**Affiliations:** ^1^Department of Psychology, Center for Social and Cultural Psychology, University of Leuven, Leuven, Belgium; ^2^Fonds Wetenschappelijk Onderzoek, Brussels, Belgium; ^3^Department of Linguistics, Multimodality, Interaction and Discourse, University of Leuven, Leuven, Belgium

**Keywords:** stereotypes, coping, stigma, workplace barriers, threat, gender, minorities

## Abstract

Despite changes in their representation and visibility, there are still serious concerns about the inclusion and day-to-day workplace challenges various groups face (e.g., women, ethnic and cultural minorities, LGBTQ+, people as they age, and those dealing with physical or mental disabilities). Men are also underrepresented in specific work fields, in particular those in Health care, Elementary Education, and the Domestic sphere (HEED). Previous literature has shown that group stereotypes play an important role in maintaining these inequalities. We outline how insights from research into stigma, social identity, and self-regulation together increase our understanding of how targets are affected by and regulate negative stereotypes in the workplace. This approach starts from the basis that members of negatively stereotyped groups are not just passive recipients of negative attitudes, stereotypes, and behaviors but are active individuals pursuing multiple goals, such as goals for belonging and achievement. We argue that it is only by understanding stigma from the target’s perspective (e.g., how targets are affected and respond) that we can successfully address workplace inequality. Key in this understanding is that stereotypes, prejudice, and discrimination have taken on much more subtle forms, with consequences for the way members of stigmatized groups cope. These insights lead us to propose an approach to understanding barriers to workplace equality that highlights four key aspects: (1) the different (often subtle) potential triggers of identity threat in the workplace for members of stigmatized groups; (2) the ways in which members of stigmatized groups cope with these threats; (3) the role of supportive factors that mitigate potential threats and affect self-regulation; and (4) potential hidden costs for the self or others of what appears at first to be effective self-regulation. The focus on threats, coping, support, and potential hidden costs helps us understand why current diversity efforts are not always successful in increasing and maintaining members of stigmatized groups in organizations and provides insight into how we can aid efforts to effectively lower barriers to workplace equality.

## Introduction

Despite changes in their representation and visibility, women and ethnic and cultural minorities are still strongly underrepresented in various work fields and higher occupational positions. Similarly, there are serious concerns about the inclusion and day-to-day workplace challenges facing LGBTQ+ people (Hebl et al., 2002), people as they age (Diekman and Hirnisey, 2007), and people dealing with physical or mental disabilities (Wilson-Kovacs et al., 2008). Men are also underrepresented in specific work fields, in particular those in Health care, Elementary Education, and the Domestic sphere (HEED; Croft et al., 2015).

The underrepresentation of these social groups is problematic as equitable representation is an indicator of the presence of equal opportunities and social justice (Eagly, 2016; Ellemers and Rink, 2016). Also, in inclusive work climates, diversity can positively affect corporate performance and team effectiveness (Gonzalez and Denisi, 2009; Nishii, 2013; Van Knippenberg et al., 2013; Ellemers and Rink, 2016), and people are more attracted to organizations perceived as concerned with justice and morality (Van Prooijen and Ellemers, 2015). There is thus a need to tackle the underrepresentation of different social groups in work contexts.

**Figure 1:**
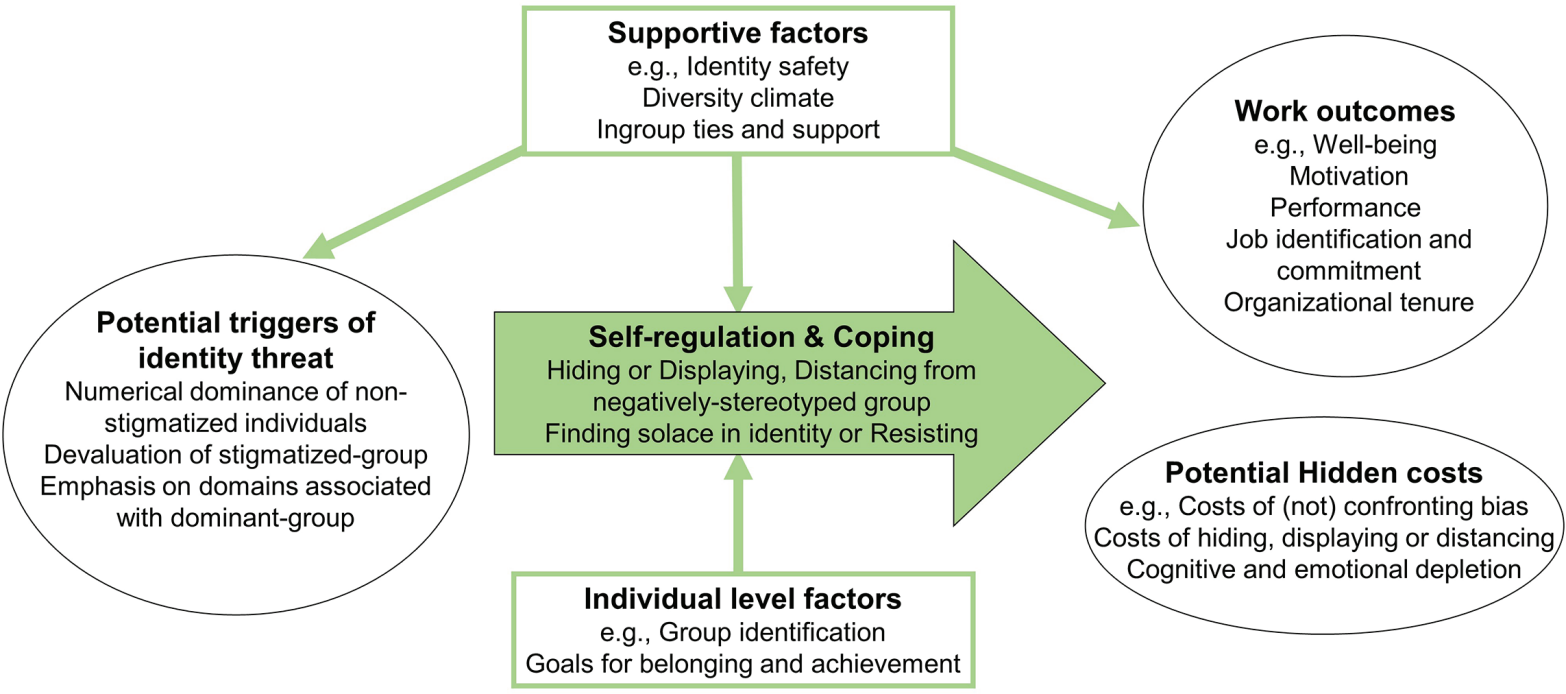
Conceptual model: a threat, support, and potential hidden costs approach to understand how members of stigmatized groups cope with workplace inequality.

Tackling underrepresentation means understanding why these inequalities continue to exist despite increased movement toward equality. We argue that to truly understand and hence successfully address workplace inequality, it is vital to know how members of underrepresented groups are affected by and respond to the workplace challenges they face. We outline a target-focused approach that integrates research on stigma and social identity with work on self-regulation. We begin with insights from research into stereotypes, prejudice, and discrimination that helps us understand the potential workplace threats members of stigmatized groups face. We then move on – from this focus on “perpetrators” of inequality and on targets as passive victims – to a focus on targets as active agents coping with stigma-related threat. Specifically, we make the case that four key aspects need to be understood to address workplace inequality (see Figure 1): (1) the different potential triggers of identity threat; (2) the ways individuals self-regulate and cope with these threats and the individual level factors that affect this; (3) supportive workplace factors that can mitigate the impact of threat; and (4) recognition of the potential hidden costs of regulating such threats.

Together these four key aspects present a base for building successful programs addressing workplace inequality. The focus on threats, coping, support, and potential hidden costs brings a more comprehensive understanding of the complexities and nuances by which stigma has its often subtle effects. It demonstrates that taking the target’s perspective into account is indispensable in effectively lowering workplace equality barriers. As such, it helps us understand why current workplace diversity efforts that tend to focus on either fixing the perpetrator or fixing the victim are not always successful in attracting and retaining members of stigmatized groups, and provides tools to effectively approach stigma in the workplace.

### A Short History of Psychological Research on Social Inequalities

To understand how individuals cope with workplace stigma, we need to first give an overview of how the field of psychology has approached the topic of social inequalities over time. For many years, research on inequalities focused on the origins of bias and discrimination. This work sought to understand why majority or high-status groups have negative attitudes, prejudices, and stereotypes, and how these can be altered to increase social equality. Major insights followed from such work into what stereotypes are, how they form and affect outcomes of members of stereotyped groups. This included insights into – and the complexities of – reducing stereotypes (for reviews, see Nelson, 2009; Dovidio et al., 2010b).

Increasingly, however, it became clear that this work on external barriers faced by members of stigmatized groups was missing an important part: an understanding of the ways members of stigmatized groups experience stigma. This emphasis came much later, from the late 1980s onward, along with the increased representation of members of negatively stereotyped groups in the field as researchers. In part through this influx, social inequalities were looked at from a different perspective, examining questions that gave a more central place to the experiences of targets of prejudice, stereotyping, and discrimination. Initially, this work focused on the target as a passive recipient, and evidenced the harm being done. This highlighted that targets can become threatened in their social identity as members of stigmatized groups, with consequences for their well-being, motivation, and performance (e.g., for overviews, Smith et al., 2007; Schmader et al., 2008; Emerson and Murphy, 2014). Increasingly, however, this work emphasized that members of stigmatized groups are not just passive recipients who in essence are waiting around to be discriminated against, but that they also respond and in this way influence outcomes. This was reflected, for example, in early key work by Crocker and Major and by Swim on the target’s perspective (Crocker and Major, 1989; Crocker et al., 1998; Swim et al., 1998; Oyserman and Swim, 2001). The research increasingly showed that members of disadvantaged groups are quite resilient to stigma and that it is too simple to assume that experiences with prejudice, stereotyping, and discrimination automatically get transformed into low well-being and negative educational or work outcomes (Barreto, 2014; Leach and Livingston, 2015; De Lemus et al., 2016).

Increasingly then, the field has begun to examine active coping with stigma and has made substantial gains in understanding exactly how these coping processes work, with this work based on three main literatures that overlap and feed into one another: first, major strides were made through research from the social identity perspective which from its earliest days focused on how group identities affect relations between groups and on how identity processes affect cognition, affect, and behavior (e.g., Tajfel and Turner, 1979; Turner et al., 1987; Ellemers et al., 1999, 2002). Research on social identities made clear that social identities are malleable (can be emphasized or deemphasized), that people are motivated to pursue a positive sense-of-self, and that this self stems in part from the social groups to which people feel they belong. When a group is valued in a given context (for instance, at work), one’s membership in this group – or social identity – can increase one’s positive sense-of-self. However, when a group is devalued (i.e., faces negative stereotypes, is discriminated against), one’s sense-of-self can become threatened. Further work noted that such social identity threats trigger targets’ responses to reduce the threat. These responses include individual mobility (e.g., attempting to acquire higher workplace status), emphasizing other, more valued qualities of one’s group (e.g., emphasizing that women bring superior interpersonal skills to the workplace), or taking collective steps to challenge the lower position of one’s group (e.g., advocacy for workplace equal opportunity policies). This clarified the important role of groups in coping with negative stereotypes, prejudice, and discrimination, and the different personal and social identity strategies people may use to protect a positive sense-of-self (e.g., Heilman, 2012).

The second literature base for strides in understanding how targets cope with negative stereotypes, prejudice, and discrimination came in the form of the stigma perspective that became increasingly merged with the social identity perspective over time (e.g., Crocker and Major, 1989; Steele and Aronson, 1995; Aronson et al., 1998; Crocker et al., 1998; Swim et al., 1998; Oyserman and Swim, 2001; Schmader et al., 2008; Shelton et al., 2010; Barreto, 2014). Early work outlined how people identify and react to prejudice in interpersonal and intergroup settings. Increasing insight was gained into the effects of stigma on targets’ assessments of their abilities, motivation, and performance and on self-esteem and well-being (Crocker et al., 1998; Swim and Stangor, 1998). Related work examined social stigma as a potential stressful event (e.g., Miller and Major, 2000; Miller and Kaiser, 2001; Miller, 2006), noting that a stress response occurs when individuals perceive a self-relevant threat that exceeds their coping resources (Miller and Major, 2000; Miller and Kaiser, 2001; Major and O’Brien, 2005). Not only several coping efforts to regulate emotion, cognition, and behavior, but also one’s own physiology and the environment were proposed as responses to stressful events or circumstances (Connor-Smith et al., 2000; Compas et al., 2001). For instance, people can cope through increased engagement (e.g., enhancing a sense of personal control, changing the way one thinks about a situation through positive thinking or cognitive restructuring), or disengagement (coping efforts that disengage from or avoid the stressor; Miller and Kaiser, 2001; Miller, 2006). The stigma and coping perspective helped understand emotional, cognitive, and behavioral coping and clarified how the same stressor may be more or less impactful and may lead to different coping responses for different people and in different situations.

Lastly, our understanding of how members of stigmatized groups respond to negative stereotypes, prejudice, and discrimination has been increasingly influenced by work from a self-regulation perspective (e.g., Carver and Scheier, 1998, 2002; Swim and Thomas, 2006; Fiske, 2008; Higgins, 2012; Inzlicht and Legault, 2014). This work takes as a premise that people actively pursue multiple goals. Every individual has core social motives that drive behavior (e.g., esteem, belonging, self-enhancement), and these can become more or less of a concern through the situation people find themselves in (Fiske, 2004; Vignoles et al., 2006; Vignoles, 2011). Self-regulation processes begin when people compare their perceptions of the current situation with their goals or standards (Carver and Scheier, 1998, 2002). A comparison that reveals a discrepancy between inputs and desired goals creates motivation to reduce the discrepancy. Applied to the workplace, the self-regulation perspective leads to the understanding that responses of members of stigmatized groups need to be examined from a goal perspective – distinguishing, for example, goals for achievement and belonging, and that – depending on which goal is salient – people may come to different responses.

Together the blending of these three sets of literature have provided a much better base to understand how individuals cope with stigma in the workplace – e.g., stereotype threat as a cost of identity threat and stigma regulation as discussed later. Also, not only did insights regarding coping with stigma develop and change over time, so did the groups being studied. Early work focused on women and ethnic minorities as the prototypical groups facing stigma. It was only much more recently – also in response to societal changes in laws, attitudes, and interests that increased the visibility of other targets – that others started to be studied, including LGBTQ+, individuals facing age-related workplace stereotypes, and individuals facing physical or mental disabilities. An important recent addition is the focus on men facing stereotypes in fields where they are underrepresented (in particular in HEED – Health care, Elementary Education, and the Domestic sphere; Croft et al., 2015; Meeussen et al., 2019). Also, recent research has begun to focus on intersectionality, examining the experiences of individuals who are members of more than one stigmatized group (Purdie-Vaughns and Eibach, 2008; Cole, 2009; O’Brien et al., 2015; Remedios and Snyder, 2018). While many of the processes of threat and coping and their consequences are shared, each group is also characterized by particular characteristics or experiences (e.g., how visible identities are, whether there is one or multiple stigmas as for female ethnic minorities, what the costs of confronting stigma are, whether a stigma broadly affects many domains or a particular domain [e.g., stigma facing ethnic minorities vs. men in HEED]). In fact, through research focusing on each of these groups, the field as a whole has gained a much more thorough understanding of threat and coping, with insights and questions particularly relevant for one group aiding insights for other groups (e.g., see Deaux and Lafrance, 1998; Creed, 2006; Wilson-Kovacs et al., 2008; Crandall et al., 2009; Herek, 2009; Hebl et al., 2010; Dovidio et al., 2010a).

Building on these research traditions described above, we outline a threat, support, and potential hidden costs approach to help understand how individuals cope with workplace prejudice, discrimination, and stereotypes. As noted, we discuss four key aspects to help address workplace inequality: (1) understanding the different potential triggers of threat; (2) understanding how individuals cope with these threats; (3) identifying the supportive factors that may minimize these threats; and (4) increasing insight into the potential hidden costs of regulating identity threat.

## Potential Triggers of Threat in the Workplace

In order to address the threats individuals face in the workplace and to counteract barriers to workplace equality, it is important to understand the different (often very subtle) factors that can trigger identity threat. Identity threat is the psychological threat arising from possible devaluation of one’s group (Branscombe et al., 1999a). While related terms are used in other literatures (e.g., stressor, demand), we use the term typical of the social identity tradition. As we outline below, workplace identity threat can result from three kinds of triggers. These triggers may be activated solely or together, and each trigger can point to different solutions to reduce workplace inequality. The first is the higher workplace numerical presence of members of the non-stigmatized group, the second the devaluation and discrimination of the stigmatized group, and the third a workplace emphasis on characteristics and domains typically associated with the non-stigmatized group (for related discussions, see Inzlicht and Ben-Zeev, 2000; Steele et al., 2002; Van Laar et al., 2010).

### The Numerical Dominance of the Non-Stigmatized Group

First, increasing evidence shows how the numerical dominance of the non-stigmatized group in the workplace can by itself already present a threat to members of stigmatized groups. This results from basic group processes: people categorize themselves and others into ingroups and outgroups based on observable similarities and differences (Tajfel and Turner, 1979; Turner et al., 1987). Being different from others along a specific dimension (e.g., gender, ethnicity, sexual orientation, age, and disability) makes that dimension more salient (Wilder, 1984) and increases the expectation that one will be viewed in terms of that dimension (Frey and Tropp, 2006). Negative stereotypes associated with that dimension then also become more salient (e.g., Heilman, 1983; Avery et al., 2008; Somvadee and Morash, 2008) and in turn affect outcomes (effects of stereotypes on outcomes are discussed in the next section).

Consistent with this, considerable work in social psychology has demonstrated effects of being in the numerical minority. Generally, environments can be perceived as more identity threatening when they contain fewer others of one’s group (i.e., when they lack critical mass; e.g., Allmendinger and Hackman, 1995; Cohen and Swim, 1995; Sekaquaptewa and Thompson, 2002, 2003; Avery et al., 2008; Duguid, 2011). When in the minority, individuals tend to become vigilant regarding the minority identity, with various negative consequences (Emerson and Murphy, 2014). Illustrative is research in STEM domains (Science, Technology, Engineering, and Math), a domain where women tend to be in the numerical minority. Research by Murphy et al. (2007) showed that women in STEM viewing a video of a STEM conference depicting a majority of men/minority of women (vs. a balanced ratio) exhibited more cognitive and physiological vigilance, reported lower belonging, and had less desire to participate in the conference (see also Richman et al., 2011). Men were not influenced by the numerical representation in this (for them) identity-safe domain. Similarly, Inzlicht and Ben-Zeev (2000) have shown that women’s (but not men’s) math performance can become impaired when in a numerical minority, but not when gender is balanced. Relational demography research has also shown numerical underrepresentation (in terms of gender, age, and ethnicity) to be associated with lower organizational commitment, lower job satisfaction, lower work motivation and performance, and increased turnover (Liao et al., 2004; Riordan et al., 2004), particularly when the numerical representation triggered increased perceived conflict between the work identity and the underrepresented-group identity (Veldman et al., 2017). Among African-American professionals, research has related numerical underrepresentation to lower well-being and stronger experiences with employment discrimination (Jackson et al., 1995; Avery et al., 2008). Also, the effects of numerical underrepresentation can be additive when someone is part of multiple minority groups (Jackson et al., 1995; Remedios and Snyder, 2018). In other words, a female leader of color in a predominantly White and masculine context is affected not only by her ethnic minority status (as one of few Blacks) but also by her gender minority status (as one of few women). Moreover, not only generally numerical-representation matters, but particularly also representation at the various (and especially higher) levels of the organizational hierarchy (Unzueta and Binning, 2012), and for some groups numerical underrepresentation is a given, due to their actual numerical minority status in society (e.g., sexual minorities, people with disabilities). Also, those with a concealable stigma have a harder time identifying others who share their stigma and may thus have an even harder time feeling there is any presence of their group.

### The Devaluation of Stigmatized Groups

A second way in which identity threat can be triggered in the workplace is through devaluation of stigmatized groups. One of the clearest cues regarding devaluation is seeing discrimination, and many studies have shown the negative effects of experiencing prejudice and discrimination, including higher stress and lower psychological well-being – and lower psychological and physical health more generally (Pascoe and Smart Richman, 2009; Schmitt et al., 2014; Jones et al., 2016).

While blatant discrimination is an obvious cue, devaluation in a given organizational context is often more likely to come from smaller subtler cues. While blatant cues explicitly display negative attitudes toward a group, subtle cues convey the same belief as blatant messages but in more covert and often unintentional ways (Dovidio, 2001; Barreto and Ellemers, 2005; Ellemers and Barreto, 2015). When such subtle cues signal an identity’s low value in a specific context, this particular group identity becomes salient for the group member, and a vigilance process is initiated. This directs stigmatized individuals’ attention toward additional cues to determine the value and meaning of their social identity in that context (Murphy et al., 2007; Murphy and Taylor, 2012). If situational cues confirm the possibility a social identity may be negatively evaluated, vigilance increases. Also, chronic and situational expectations about being stigmatized increase attention to identity-relevant cues (Kaiser et al., 2006). In fact, a single subtle cue can trigger experiences of social identity threat even if the setting exhibits no overt evidence of prejudice or discrimination (Murphy et al., 2007). Also, one cue can determine the interpretation of another (ambiguous cue) in both positive and negative directions (Kaiser et al., 2006).

One of the strongest demonstrations of contextual cues triggering these effects comes from the extensive stereotype-threat literature. Hundreds of studies have now shown that cues making salient negative group stereotypes trigger concern about being judged on the basis of these stereotypes (Steele et al., 2002). In fact, the stereotypes may even only exist in the mind of the stigmatized group member: for stereotype threat to occur, others around one do not need to hold a negative stereotype of the group, one only need to believe that they do. This concern can set in motion anxiety, mind wandering, negative thinking, and a wish to disprove the stereotype. These together co-opt working memory, resulting in decreases in performance and lower well-being (e.g., Steele and Aronson, 1995; Blascovich et al., 2001; Steele et al., 2002; Schmader and Johns, 2003; Cadinu et al., 2005; Johns et al., 2008; Schmader et al., 2008; Inzlicht and Schmader, 2012; Mendes and Jamieson, 2012; Schmader and Beilock, 2012; Pennington et al., 2016; Spencer et al., 2016). Research in organizational contexts has shown that experiences with stereotype threat negatively affect career aspirations, career confidence, and professional identification (see Kalokerinos et al., 2014 for an overview). Also, the effects of stereotype threat have been shown in all kinds of groups. For example, stereotype threat has been related to more negative job attitudes and increased turnover intentions among female employees in the legal profession (Von Hippel et al., 2011); to higher turnover intentions among male primary school teachers (Kalokerinos et al., 2017); and to more negative job attitudes, poorer work mental health, and increased intentions to resign among older employees (von Hippel et al., 2013).

Information as to whether a certain identity is devalued can come from various types of cues. For example, workplace cues that make a specific identity and accompanying stereotypes salient (e.g., when physical access to important company locations is difficult for employees in a wheelchair) or from cues that signal the (under)representation of a stigmatized group (e.g., company photos showing only White males). Devaluation can also come from more general cues that signal an organization’s diversity beliefs and values (e.g., value for meritocracy that may fail to acknowledge structural inequalities) or from organizational structures and policies (e.g., colorblind policies that may fail to recognize the existence and value of different cultural identities; see Emerson and Murphy, 2014). Numerous studies have shown the negative effects of such workplace cues. For instance, when objects in a computer science environment were stereotypically male, women were less interested in computer science and felt less of a sense of belonging (Cheryan et al., 2009; see also Murphy et al., 2007). Similarly, Hall et al. (2015, 2019) showed among female engineers that low acceptance cues from others in daily conversations (rather than explicitly hostile cues) led to a sense of identity threat, which in turn increased mental exhaustion and disengagement (see also Ahlqvist et al., 2013). Other studies have shown similar negative cue effects, for example, lowered leadership aspirations in women following exposure to gender stereotypic advertisements (Davies et al., 2005). Also, Avery et al. (2007) showed that organizational cues indicating low value for diversity predict higher absenteeism in African-American employees. Similarly, Purdie-Vaughns et al. (2008) showed that cues suggesting low minority representation coupled with cues suggesting the organization values colorblindness (vs. values diversity) led to higher identity threat and workplace distrust in African-American professionals.

This work shows that devaluation often stems from small subtle triggers that have profound effects, an understanding key to addressing devaluation in the workplace.

### Emphasis on Domains Associated With the Dominant Group

A third way in which threats can present themselves to members of stigmatized groups is through the workplace emphasis on domains perceived to describe the non-stigmatized group more than the stigmatized group (Derks et al., 2006, 2007a). This can result in lower perceived fit and a threatening environment for members of stigmatized groups, leading to lower well-being, lower motivation and disengagement, and lower performance and higher turnover intentions.

Research on role congruity has shown, for example, how emphasis on domains or characteristics perceived to better fit the non-stigmatized than one’s stigmatized group may trigger identity threat (Heilman, 1983; Eagly and Karau, 2002; Lyness and Heilman, 2006; Bongiorno et al., 2014). Much of this work has been conducted with regard to gender. Thus, in traditionally masculine professions such as STEM domains, the police force, or the military, characteristics traditionally associated with men are more strongly valued than characteristics traditionally associated with women (Somvadee and Morash, 2008; Archbold et al., 2010; Cheryan et al., 2017). However, perceived role (in)congruity affects other groups and intersectional identities, too: e.g., older employees – stereotyped as rigid and unadaptable – are at a disadvantage in rapidly changing work domains (Diekman and Hirnisey, 2007), and employees with a mental illness may experience added prejudice when their mental illness is seen as stereotypical of the other gender (Koenig and Eagly, 2014).

Other research has also shown that there can be a mismatch between qualities, values, or norms that tend to be associated with members of traditionally underrepresented groups and the settings they are entering and that this is subtly signaled in the context (Stephens et al., 2012a,b, 2014; Schmader and Sedikides, 2018; Veldman et al., 2019). For example, Gaucher et al. (2011) showed that this may occur for women through job descriptions that use more masculine-themed words (e.g., emphasizing dominance, competitiveness). Men too are perceived as not fitting HEED domains that emphasize traditionally female qualities such as being nurturing, helping others, and being emotionally involved (Cejka and Eagly, 1999; Fiske et al., 2002; Wayne and Cordeiro, 2003; Rajacich et al., 2013; Rudman and Mescher, 2013).

### Conclusions Regarding Potential Triggers of Threat in the Workplace

In summary, three kinds of triggers may elicit threat for members of stigmatized groups at work, and each trigger may call for different solutions to reduce workplace inequality (we discuss these in the implications section). First, members of stigmatized groups tend to be in the numerical minority in the workplace. Being in the minority and dissimilar from others tends to make that social identity salient, with negative consequence for well-being, motivation, and performance. Second, devaluation – often through subtle small environmental cues – can profoundly affect the outcomes of members of negatively stereotyped groups. Third, the emphasis on domains stereotypically associated with the dominant group can create an expectation of underperformance and an unwelcoming work environment for members of stigmatized groups. As this overview shows, the group identities themselves are not the problem: it is the threat that comes along with that identity in particular contexts and in various ways that can result in negative consequences for well-being, motivation, turnover, and performance. Also, all of these factors tend to work together: workplace underrepresentation sends the message that the reasons for the underrepresentation of a particular group are legitimate – the result of the lower abilities or skills on the part of these individuals. This bolsters devaluation and maintains segregated roles and contexts that themselves then reconfirm the stereotypes.

While the above research identifies the various potential triggers of threat and the substantial effects of these threats for members of stigmatized groups, research has also brought a much better understanding of the intricate ways that members of negatively stereotyped groups have found to cope with these threats. We turn to this issue next.

## How People Cope With Stigma-Related Threat

As discussed, the current understanding of stigma reflects members of stigmatized groups not as passive recipients of stigma-related threats but as active actors pursuing multiple goals in the workplace and beyond (Fiske, 2004, 2008; Swim and Thomas, 2006). Although identity threat can threaten various goals, two key ones are the goal to achieve (to feel competent, to do well) and the goal to belong (to fit in, to feel at home; Steele et al., 2002; Barreto, 2014; Hall et al., 2015). Potential threats to these goals trigger self-regulatory processes and coping, with people adjusting behavior, cognition, and affect to try to achieve these goals (Carver and Scheier’s 1998, 2002; Affect-Alarm Model of Self-Control – Miller and Major, 2000; Inzlicht and Legault, 2014). Workplace threats can differentially affect these specific goals and in turn trigger different regulatory responses that can move people in different directions (Steele et al., 2002; Swim and Thomas, 2006). Thus, concerns for achievement may lead members of stigmatized groups to try even harder to overcome doubts surrounding their group membership. Alternatively, people may disengage or exit if they perceive they cannot change others’ attitudes, or if the challenge is too great, too stressful, or simply too aversive (see also Wrosch et al., 2003). Concerns for belonging meanwhile may lead people to focus on social relations: attempting to increase their fit with others, seeking solace in their shared identity with similar others at work, or working together with these others to challenge workplace barriers. Also here, concerns for belonging may lead members of stigmatized groups to exit the environment and seek environments with increased belonging.

These goals for achievement and belonging need to be understood in the context of the modern workplace where forms of bias have taken on much more subtle, harder to recognize forms (Dovidio, 2001; Barreto and Ellemers, 2005; Cortina, 2008; Ellemers and Barreto, 2015). Blatant forms of bias and discrimination are increasingly less acceptable – so while they are more easily protested against than in the past, they are also much less pervasive (see also Operario and Fiske, 2001). Instead individuals have to face more subtle cues, leaving them unsure whether in fact discrimination or devaluation occurred (e.g., Crosby et al., 1993; Williams et al., 2003; Sterk et al., 2018) – which may make responses that do not involve claiming bias or collectively protesting more likely in the workplace (Wright et al., 1990; Becker, 2012; Branscombe et al., 2012). We address a number of these responses here, varying from hiding and concealing stigmatized identities to finding solace in one’s group and resisting.

### Hiding, Displaying, and Distancing

One of the ways members of stigmatized groups may deal with identity threat is through attempting to acquire, display, or emphasize the qualities they perceive to be important or valued in the context and hide or conceal those that are not. Individuals alter their self-presentation in these ways to try and avoid bias and rejection by coworkers and to increase their belonging (an assimilation strategy – Garcia and Crocker, 2008; Newheiser and Barreto, 2014; Newheiser et al., 2017). People may emphasize or display the qualities that they believe to be most fitting in the context (for instance, ethnic minority employees emphasizing ethnic majority characteristics, or women in leadership positions emphasizing agentic characteristics; Derks et al., 2011a,c, 2015; see also earlier discussion on workplace emphasis on domains associated with the dominant group). Additionally, individuals may hide or conceal their threatened identities. For instance, individuals have been found to hide (vs. reveal) concealable stigmatized identities, such as LGBTQ+ identity, having a history of mental illness, or poverty (Newheiser and Barreto, 2014). Similarly, Pronin et al. (2004) showed that women strongly identified with math disavowed traditionally feminine characteristics strongly associated with – but not those that weakly associated with – the gender-math stereotype.

People often combine hiding and displaying in “self-group distancing” as an identity management strategy. Specifically, upwardly mobile members of negatively stereotyped groups may increasingly distance themselves from their negatively stereotyped group in the workplace (Pronin et al., 2004; Derks et al., 2011a,c, 2015, 2016; Becker and Tausch, 2014; Faniko et al., 2017). This can occur inadvertently – as the individual tries to best fit the environment dominated by members of the non-stigmatized group – or more strategically, when upwardly mobile individuals recognize that presenting the self in ways more acceptable to the non-stigmatized group may bring certain benefits (e.g., being perceived as a potential future leadership candidate) or avoid costs (e.g., avoid restrictions on access to key social networks). A number of indicators of self-group distancing have been found, including an increased emphasis on one’s outgroup characteristics, emphasizing that one is different from other members of one’s stigmatized group, concealing the devalued identity, increasing the expression of stereotypical views of other members of one’s group, and denying the existence of bias against one’s group (Ellemers et al., 2004; Pronin et al., 2004; Burkley and Blanton, 2008; Derks et al., 2011c, 2015, 2016; Becker and Tausch, 2014; Faniko et al., 2017). Such self-group distancing behaviors can be mild (e.g., Gay employees not objecting when stereotypes about LGBTQ+ are voiced in meetings) to more major (e.g., a female employee saying that the underrepresentation of women in the company is an indication that women simply do not have what it takes to excel).

When women show self-group distancing behavior, this has been referred to as “Queen bee” behavior. Women have indeed been found to show self-group distancing behavior in response to existing stereotypes, prejudice and discrimination in their organizations (Pronin et al., 2004; Cohen and Garcia, 2005; Derks et al., 2011a,c, 2015; Kaiser and Spalding, 2015). For instance, senior policewomen showed more self-group distancing following reminders of gender bias at work (Derks et al., 2011c). Research has also shown that individuals who are less identified with their stigmatized group are more likely to self-group distance under threat, and indeed high identifiers may not show distancing at all (e.g., Derks et al., 2009, 2015; Kaiser and Spalding, 2015). This is consistent with other work within the social identity approach showing that low identifiers may be less loyal and faithful to the group as threats increase, while high identifiers are more likely to stay loyal and choose collective routes to address inequality (Derks et al., 2009; Hersby et al., 2009; Iyer and Ryan, 2009; Ellemers and Van Laar, 2010). As such then, self-group distancing appears to be an identity management strategy aimed at benefitting individual mobility and individual-level outcomes (Tajfel and Turner, 1979; Derks et al., 2007b, 2016).

However, self-group distancing occurs not only for women but also for other groups (see Derks et al., 2016 for reviews). Older adults, for example, respond to stigma with reduced group identification, and by indicating that they feel younger than they are (Weiss and Lang, 2012). Also, following priming with ethnic bias, ethnic minorities present themselves in ways fitting the ethnic majority group (Derks et al., 2015), and contact with the majority group increases the likelihood that ethnic minorities will distance (Becker et al., 2013). Also, Gay men have been found to distance themselves from the stigma of the “feminine” homosexual by emphasizing their masculinity and rejecting Gays they see as stereotypically “feminine” (Eguchi, 2009; Hunt et al., 2016). Even in minimal groups created in laboratory settings, being undervalued and underrepresented induces self-group distancing (Wright and Taylor, 1999).

Hiding, displaying, or distancing can be effective to the degree that this presentation of the self is accepted by the various workplace parties. Also, hiding one’s identity is possible to the degree that identities are concealable (e.g., low SES, sexual orientation, mental illness), and less easy for visible identities such as gender or ethnicity (Quinn, 2017). Acquiring, displaying, or emphasizing qualities typical of the dominant group meanwhile is more generally available to all kinds of groups. However, this may not always be accepted by other members of one’s stigmatized group (Marques and Paez, 1994; Van Laar et al., 2014), or may lead to rejection by members of the dominant group who do not accept the altered presentation. This has been shown, for example, in the case of women showing more agentic (and lower communal) traits and behaviors, and as a result being rejected by both men and women (Fiske et al., 1991; Rudman and Glick, 2001; Gabriel et al., 2017). Similarly, men are not always accepted, welcomed, or valued when showing more communal qualities, for example, in HEED domains (Lockwood and Kunda, 1999; Wayne and Cordeiro, 2003; Lockwood, 2006; Bell-Scriber, 2008; Rudman and Mescher, 2013). Lastly, various personal and group costs may result from self-group distancing, hiding, and concealment, as we will see later in the section on potential hidden costs.

### Finding Solace in Identity or Resisting

While members of stigmatized groups may try to hide, conceal, or minimize their threatened identity, emphasize their outgroup characteristics, or more generally distance from their stigmatized group, they may also go in the opposite direction: finding solace in strong group identities that they share with others, or resisting prejudice, stereotypes, and discrimination (Branscombe et al., 2012).

Research on identity affirmation has shown that strong group identities can indeed bring solace. This work is grounded in extensive research on the function of group identities, with group identities providing a base for self-definition, allowing individuals to maintain their distinctiveness, and to enhance positive views of the self (Spears et al., 1997; Ellemers et al., 2002; Jetten et al., 2014). Specifically, this work shows that members of stigmatized groups benefit when they themselves (or the surrounding context, as outlined later under “supportive factors”) value or affirm positive aspects of this identity (Biernat et al., 1996; Sherman and Cohen, 2002; Sherman et al., 2007; Oswald and Chapleau, 2010; Ghavami et al., 2011; Latrofa et al., 2012). Research has shown that members of stigmatized groups indeed personally affirm group identity in response to threat (Derks et al., 2006, 2007a; Latrofa et al., 2012; see also Crocker and Major, 1989), and that this identity affirmation helps against stereotype threat (Logel et al., 2009), buffers self-esteem (Spencer-Rodgers et al., 2016), and protects motivation and performance (Derks et al., 2006, 2007a), in part by decreasing physiological threat and increasing physiological challenge (Derks et al., 2011b). Such affirmation of group identities is particularly likely to be shown by those more highly identified with their group (Derks et al., 2009; Hersby et al., 2009; Iyer and Ryan, 2009; Ellemers and Van Laar, 2010). Work on the rejection-identification model has similarly shown that rejection may motivate a return to the group (Branscombe et al., 1999b – but see Begeny and Huo, 2017, 2018 for potential costs of identification through cognitive saliency).

Members of stigmatized groups may also obtain extra motivation precisely from the stereotypes they face, trying extra hard to show the stereotypes are wrong (e.g., Steele et al., 2002; Keller, 2007; Grimm et al., 2009; Ståhl et al., 2012a; Leach and Livingston, 2015). Research using psycho-physiological indices (Blascovich et al., 2000) has shown that people indeed can resist negative effects of stereotypes, showing efficient mobilization of energy and turning threat experiences into a challenge to perform well despite negative stereotypes (Derks et al., 2011b). For example, research with women with high leadership efficacy found increased leadership identification on confrontation with gender stereotypes about leadership. They also performed better in a leadership task, despite this being physiologically stressful for them (Hoyt and Blascovich, 2007, 2010). Additionally, research has shown that when experiencing gender inequality, higher group identification may prompt resistance, resulting in greater activation of reversed gender stereotypes, stronger leadership aspirations, more persistence in stereotypically masculine domains, and greater support for collective action (De Lemus et al., 2013, 2015; Leicht et al., 2017; Van Breen et al., 2018).

Research shows that people usually do not start with resistance. Often they first try to work within the system, adapting to the new situation and trying harder, using affirmations or other ways of coping, and only when this is not effective turning to the stigmatized group to try to work together (Wright et al., 1990; Boen and Vanbeselaere, 2000; Iyer and Ryan, 2009; Branscombe et al., 2012). As discussed earlier, such direct collective responses may be less likely in modern workplaces where bias is perceived as being in the past, and less identifiable and pervasive. Altogether then, there is substantial evidence that people may find solace in strong group identities, and the sharing of these identities with others of their group, and may also find strong motivation precisely from the stereotypes they face.

### Conclusions Regarding Coping With Threat

In summary, research on coping has provided increasing insight into the various ways in which members of stigmatized groups deal with identity threat. As workplace bias has taken more subtle and ambiguous forms in many societies, more indirect responses to stigma (such as hiding, displaying, and distancing from the group, or conversely finding solace in identity and resisting the group stereotypes) are also more likely. Members of negatively stereotyped groups may minimize or conceal the threatened identity in the workplace in an effort to fit in, triggering the identity as little as possible in the minds of those with whom they interact. They may try extra hard to do well, to show the stereotypes are wrong, obtaining extra motivation precisely from the stereotypes they face. When such efforts appear insufficient, individuals may disengage from the domain altogether in an effort to protect well-being, and instead focus on domains in which they expect they may be more successful.

Of course, even though members of stigmatized groups are active agents coping with threat, this does not mean that the responsibility to do so must (only) lie with them (Ellemers and Barreto, 2015). Considerable research has provided insights into the factors in the work environment that may mitigate the effects of identity threat, and how this can be useful to organizations in their efforts to reduce workplace inequality. We discuss these contextual supportive factors next.

## Supportive Factors That Mitigate Threat or Its Effects

Increasing evidence is providing a better understanding of how supportive factors outside the individual may help members of stigmatized groups cope with identity threat in the workplace. Specifically, as shown in Figure 1, supportive factors can affect whether potential triggers of identity threat are in fact experienced as threats; affect the self-regulation and coping strategies that are available to members of stigmatized groups; and can directly affect the outcomes of members of stigmatized groups. We review recent work on identity safety and diversity climate factors (including colorblind vs. multiculturalist approaches), plus the importance of ingroup ties, role models and support for members of stigmatized groups.

### Identity Safety and Diversity Climate

An important way to reduce identity threats or the consequences of identity threat experienced by members of negatively stereotyped groups in work settings is through the creation of identity safety. Identity safety makes it less likely that identity threat is triggered. Also, identity-safe environments reduce the need to regulate any threats and directly affect outcomes. This research emerges from a number of different angles: for instance, work with groups that are in conflict or that differ in power has shown that feeling one’s group is accepted is a prerequisite for members of low status or negatively stereotyped groups to move toward reconciliation (Shnabel et al., 2009; Saguy and Kteily, 2014). Also, identity safety can be effectively created through contextual identity affirmations, signaling that a social group is valued within this organization. Affirmation of identities of importance to members of underrepresented groups has been found to protect well-being, motivation, and performance (Derks et al., 2006, 2007a, 2009; Van Laar et al., 2010). For example, contextual identity affirmation has been found to lower identity threat among those of low socio-economic status (Stephens et al., 2015), and to lower identity threat and increase well-being, motivation, and perceptions of opportunity in the workplace among young Muslim women (Van Laar et al., 2013).

One key way in which identity safety is communicated is through the diversity climate of an organization. The diversity climate signals the extent to which the workplace is open to various social groups (Huo and Molina, 2006; Purdie-Vaughns et al., 2008; Gonzalez and Denisi, 2009; Plaut, 2010). For members of stigmatized groups, perceiving a positive diversity climate that accepts, respects, and values their group helps reduce threat, relates to feeling more included, stronger organizational identification and commitment, and lower turnover intentions (e.g., Luijters et al., 2008; Gonzalez and Denisi, 2009; Plaut et al., 2009; Choi and Rainey, 2013; Podsiadlowski et al., 2013; Meeussen et al., 2014; Van Laer, 2018). Interestingly, diversity climates have effects beyond the groups specifically targeted: men of color, for instance, experience identity safety from organizational diversity policies aimed at women, and women from ethnic diversity policies (Chaney et al., 2016).

A positive diversity climate in a work organization is not sufficient if numerical underrepresentation, presence of negative stereotypes and devaluation, and the emphasis on domains associated with the dominant group outlined earlier are not addressed. A recent study showed, for example, that among women in the police force, experiencing a positive diversity climate only partially reduced the negative effects of underrepresentation, with the women continuing to show negative consequences of underrepresentation on identity conflict (Veldman et al., 2017). Other work has shown the dangers of piecemeal diversity initiatives and “token” minority representation (i.e., representation of only a few minority group members) that can blind people to existing inequality (Brady et al., 2015; Kirby et al., 2015; Anisman-Razin and Saguy, 2016; Gündemir and Galinsky, 2017), and should therefore not constitute the sole strategy to advance equality (see also Hentschel et al., 2013). Also, in generating a positive diversity climate, it is important that organizations also pay attention to the needs of the majority or high-status individuals, who similarly use information on diversity climate as an indicator of the degree to which their identity is accepted. They may resist diversity efforts within an organization when they feel that these put their group at a disadvantage (Avery et al., 2013). Thus, a focus on the value of differences in multiculturalism may be interpreted by majority members as a lack of value for their “standard” identity (Stevens et al., 2008; Plaut et al., 2011). Instead, researchers identify an all-inclusive multicultural climate or a multicultural meritocracy as most effective, with these focusing on identity safety not just for members of negatively stereotyped groups, but making sure that members of dominant or majority groups also feel valued and included (Stevens et al., 2008; Emerson and Murphy, 2014; Gündemir et al., 2017; see also Ellemers and Rink, 2016).

One aspect of a positive diversity climate that serves as an indicator of identity safety and an antidote to workplace threats for stigmatized groups is organizational support. Support can be proximal or distal, and can come in the form of instrumental support that provides tangible help to solve a problem or issue, or in the form of emotional support, offered through empathy and caring (Cohen and Wills, 1985; Wills, 1985; House et al., 1988; Richman et al., 2011; London et al., 2011a,b). As discussed earlier, possessing a social stigma in the workplace is a potentially stressful event (Miller and Major, 2000; Miller and Kaiser, 2001), and support can increase the perceived resources to cope with the stressor, thus even turning threat into positive challenge (Lazarus and Folkman, 1984a,b; Cohen and Wills, 1985; Miller, 2006). For members of stigmatized groups, experiencing support in the organization – or perceiving that it is available – predicts stronger engagement and better achievement outcomes (Eccles, 1994; Walton and Cohen, 2007; Hartman and Hartman, 2008; Richman et al., 2011; Baysu et al., 2014). Such support can come from various sources: e.g., support from representatives of the majority high-status group in the workplace can lower negative effects of stigma-related threat, and support may be especially important from those in positions of authority or power (e.g., Baruch-Feldman et al., 2002; Drury and Kaiser, 2014). Support from both these sources signals acceptance and value for the stigmatized group, and can create new norms throughout an organization, particularly in organizations where members of stigmatized groups are underrepresented, face strong negative stereotypes, or where domains associated with the dominant group are more strongly emphasized. Hall et al. (2015) showed this in their recent daily-diary study among female engineers, where having positive work conversations with male colleagues cueing acceptance protected the women from identity threat. Importantly, such positive conversations were more likely to occur in organizations perceived to have more gender-inclusive policies (i.e., in identity-safe organizational cultures; Hall et al., 2018). Recent work is more generally beginning to address support for diversity by members of high-status groups, examining the conditions under which members of high-status groups may offer support, for example as allies, and the effects this support can have (e.g., Saguy et al., 2008; Fingerhut, 2011; Becker et al., 2013; Saguy and Dovidio, 2013; Cihangir et al., 2014; Drury and Kaiser, 2014; Brown, 2015; Simon and O’Brien, 2015; Droogendyk et al., 2016; Emina et al., 2018; Good et al., 2018). In our own work, we are examining, for example, support in military and police organizations, examining whether men become more aware of gender inequality in their organization through contact with women, and effects of this contact on their support for gender-related social change.

### Importance of Ingroup Ties and Support

In lieu of or in addition to identity safety or support from the high-status majority group in the workplace, ingroup support can also provide a resource to mitigate threat. Obtaining workplace ingroup support can become more difficult if one’s group is underrepresented and if the stigma is concealed or not visible. However, ingroup support outside the organization can then offer additional possibilities. Positive effects of a connection with the ingroup when under threat is predicted by various social-psychological models, including the rejection-identification model (Branscombe et al., 1999b), and the stereotype inoculation model which shows ingroup members to function as “social vaccines,” who inoculate and strengthen fellow group members (Dasgupta, 2011), and backed up by substantial evidence (e.g., Correll and Park, 2005; Haslam et al., 2005; Bakouri and Staerklé, 2015; see also Richman et al., 2011 – but see Begeny and Huo, 2017, 2018 for potential negative effects of increased cognitive salience following identification). People are particularly likely to seek ingroup support when identity threat is high or pervasive (Branscombe et al., 2012). Such support helps individuals overcome various negative effects of threat (e.g., Cohen et al., 2000; McLeroy et al., 2001; Ostberg and Lennartsson, 2007; Rosenthal et al., 2013), increases psychological well-being, and decreases distress (Turner, 1981; Haslam et al., 2005). Ingroup support may also encourage people to pursue (rather than avoid) activities in which they are negatively stereotyped. Men, for instance, are more likely to increase engagement in HEED domains and increase HEED occupational aspirations when told that other men support and value communal characteristics (Van Grootel et al., 2018). Even through mere presence of similar others, support can lift self-esteem, improve mood (Frable et al., 1998), and provide a buffer for social identity threat (Levin et al., 2006; Richman et al., 2011).

A particular case of ingroup support comes through support from ingroup leaders and role models (see also, Unzueta and Binning, 2012). For members of stigmatized groups, demographic similarity with supervisors (e.g., ethnic or gender similarity) is related to reduced absenteeism and tardiness, and increased intent to remain in the organization (Avery et al., 2012). Also, seeing examples of successful members of one’s stigmatized group has been found to improve self-evaluations and performance, give inspiration and proof others can do it, and increase aspirations and motivation (Lockwood and Kunda, 1999; Marx and Roman, 2002; McIntyre et al., 2003, 2005; Lockwood, 2006; Marx et al., 2009; O’Brien et al., 2016; Dennehy and Dasgupta, 2017). For men too, having male role models in HEED domains increases interest in elementary teaching and nursing (e.g., Cochran and Brassard, 1979). Recent work by Morgenroth et al. (2015) suggests that role models have three distinct functions: acting as behavioral models, representing the possible, and being inspirational.

### Conclusions Regarding the Effects of Supportive Factors in Work Environment

In conclusion, research on identity safety, diversity climates, and ingroup and outgroup support suggests various ways in which supportive factors in the workplace may buffer threat or help cope with threat. Supportive factors may also moderate which self-regulation and coping are available and used to deal with threat (and this would be an interesting avenue for future research). For instance, the presence of supportive (minority) networks in the organization makes it easier to display and find solace in an identity, and to show resistance when identity-threatening experiences do occur. Meanwhile, hiding and distancing are more likely when such supportive networks or positive diversity climates are not available. This – and the work reviewed above – also highlights the benefits for members of stigmatized groups to maintain their links with other members of their group for the protection of well-being, motivation, and performance in the workplace. In addition, support from the dominant group may be key as majority individuals are still more likely to be in positions of power and seen as legitimate sources of workplace information (Drury and Kaiser, 2014). Hence, ingroup and outgroup support processes can contribute to efforts to address workplace inequality.

## Potential Hidden Costs of Dealing With Stigma

As outlined above, we have quite good understanding of the potential threats facing members of stigmatized groups in work settings. While the threats can be significant, we also know that individuals have various coping strategies at their disposal, and environments can offer important sources of support. Nevertheless, dealing with stigma – even if seemingly effectively – can have important unintended and hidden costs, either for the stigmatized individuals themselves or for other members of their group. These costs are often not at all obvious, and understanding these potential costs is important to effectively address workplace equality in the long run. Below, we discuss costs that can be particularly consequential in the workplace: costs of (not) confronting bias, costs of concealing identity and distancing from one’s group, and cognitive and emotional depletion following stigma regulation.

### Costs of (Not) Confronting Bias

One of the hardest tasks members of stigmatized groups face is deciding whether to confront or not confront the injustice they experience in the workplace, because confronting others with claims of bias or discrimination entails risks. Research has shown that members of stigmatized groups who confront bias are less likely to be believed and are evaluated less favorably than members of majority groups addressing the same bias. This is true even when the bias is acknowledged and blatant (e.g., Swim and Hyers, 1999; Czopp and Monteith, 2003; Kaiser and Miller, 2003; Czopp et al., 2006; Kaiser, 2006; Shelton et al., 2006; Rasinski and Czopp, 2010; Becker et al., 2011, 2014; Eliezer and Major, 2012; Gervais and Hillard, 2014). Also, given that bias cues have become more subtle, these costs can become even higher as the legitimacy of bias attributions is more ambiguous.

Deciding whether to confront or not can thus be a very difficult decision, and members of stigmatized groups may ruminate extensively on what is best. Even when they do not confront, this rumination about whether they should have confronted can last long after the situation has passed. Also, when they do not confront, members of stigmatized groups can face costs, such as guilt or shame about not confronting injustice, or feeling they have let down or sold out the group (Shelton et al., 2006). Also, non-confrontation may leave members of stigmatized groups feeling inauthentic, feeling they failed to be loyal to their true selves and personal goals. Such dissonance has been found to be so aversive that people who do not confront sometimes minimize the seriousness of the bias claim to restore a positive sense-of-self (Rasinski et al., 2013).

### Costs of Hiding, Displaying, and Distancing

Potential costs can also result from coping strategies that involve hiding threatened identities; emphasizing outgroup characteristics; and from distancing from the negatively stereotyped group more generally. These costs can be incurred by the self as well as by other ingroup members.

First, hiding, concealing, or distancing from an identity in the workplace can be a costly strategy for the self. Individuals hide, conceal, and distance because they believe others will view them more favorably when they minimize their stigmatized identity, and that they will thus be less likely to experience bias or discrimination (Quinn, 2017, 2018). Also, they believe that distancing from the stigmatized identity will increase their chances for acceptance and belonging (Newheiser and Barreto, 2014). However, research has shown that concealment often tends to have the opposite effect: leading individuals to feel lower belonging and acceptance (Newheiser et al., 2017). This is driven in part by people reducing their self-disclosure also of other self-relevant information beyond the stigmatized identity, and by feeling less authentic in interactions (Newheiser and Barreto, 2014). Also, research has shown that hiding (vs. revealing) a stigmatized identity is detected by external observers and by non-stigmatized interaction partners, who have less positive impressions of the person, and of the interaction, when the person conceals an identity (Newheiser and Barreto, 2014). Moreover, as with failure to confront, members of stigmatized groups may feel disloyal to their ingroup following distancing (Goldman and Kernis, 2002; Shelton et al., 2005). Distancing also lowers opportunities to obtain support from the ingroup, further increasing negative consequences for the self (Branscombe et al., 1999b; Haslam et al., 2005; Van Laar et al., 2014; Derks et al., 2016). Such findings show that distancing from one’s stigmatized identity in an organization can be a costly strategy.

Not only the self, but others too may experience costs from hiding or distancing. Recent research suggests that self-group distancing behavior by women in leadership positions has harmful effects for junior women exposed to this behavior (Sterk et al., 2018). Behaviors such as the denial of gender bias and expressions of negative views of women may be taken at face value when shown by a woman, while seen as bias when shown by a man (see also Ni and Huo, 2018). As members of one’s own group are often assumed to have positive intent toward the ingroup (Hornsey et al., 2002; Hornsey and Imani, 2004), these expressions may remain unchallenged and not counter argued by the self, and in this way affect self-evaluations and well-being (Barreto and Ellemers, 2005; Sterk et al., 2018; c.f., Ni and Huo, 2018)^1^. Thus, while self-group distancing can allow leaders who are members of stigmatized groups to cope with experienced threats, it may increase negative consequences for subordinates coming up in the ranks.

Other work has shown that distancing behaviors may have more general negative effects for addressing workplace equality. While members of stigmatized groups are unlikely to see self-group distancing behavior as bias, it is even less likely that members of the dominant group will do so. People tend to believe that representatives of groups have their groups’ interests at heart (Sutton et al., 2006) – and thus members of stigmatized groups expressing stereotypical views of their own group (e.g., as having lower abilities or being less committed), or denying the existence of discrimination against their group, may be perceived as presenting the objective truth as to the current degree of inequality. This has important ramifications, as members of dominant groups can play a valuable role in addressing workplace inequality. Hence, an important avenue for future research is to examine to what extent members of non-stigmatized groups indeed start to believe inequality is less of a problem when successful members of stigmatized groups (e.g., female leaders) deny the existence of discrimination and express negative stereotypes of their own group.

Also in other ways, distancing has costs for the organization at large: members of stigmatized groups trying to hide, fit in, and assimilate into the organizational context undermines the organization’s potential to profit from diversity (e.g., see Ellemers and Rink, 2016). Again then, distancing from the group – like other responses to threat such as exiting when one feels low fit – reduces the likelihood that organizations will actually change to become more welcoming to members of stigmatized groups. While distancing behaviors result from social inequality, they can then also contribute to the maintenance of workplace inequality.

### Cognitive and Emotional Depletion

A third set of potential costs of coping with stigma is cognitive and emotional costs for the individual. As described earlier, coping with threat can at first boost energy and resources: individuals often try extra hard to overcome stereotypes and recruit extra resources to do so (Hoyt and Blascovich, 2007, 2010; Ståhl et al., 2012a). In fact, the prevention focus or vigilance that accompanies stereotype threat may be especially useful to recruit such resources (Seibt and Förster, 2004; Koch et al., 2008, 2009; Ståhl et al., 2012b; see also van Peer et al., 2007; Putman and Roelofs, 2011) and to more effectively differentiate (and thus choose) between signals and environments offering threat versus safety (Seligman, 1971; Öhman and Mineka, 2001; Ståhl et al., 2012b). However, coping with workplace stigma has many aspects: constant vigilance for threat, especially among those highly identified with their stigmatized group (Begeny and Huo, 2017, 2018); managing and suppressing stereotype-relevant thoughts and feelings; effectively negotiating threatening contexts; choosing to confront or not confront bias; avoiding mistakes and the confirmation of group-relevant stereotypes; and more generally regulating threat (emotional coping, accepting, or resisting). To some degree, targets can become better at – and habituated to – responding to stigma, such that those who have more frequent experiences and practice dealing with stigma become better at doing so and suffer fewer cognitive costs (Crisp et al., 2009; Johnson et al., 2010). Nevertheless, all of these aspects take cognitive and emotional energy and can eventually lead to exhaustion (e.g., Schmader and Johns, 2003; Johns et al., 2008; Logel et al., 2009; Ståhl et al., 2012a; Hall et al., 2015; c.f., Baumeister et al., 1998; Muraven and Baumeister, 2000). Research has shown that this exhaustion has negative consequences for later similar tasks as well as for other domains (e.g., reducing regulation of learning behaviors, lowering persistence on physical tasks, and increasing unhealthy eating behavior – for an overview, see Schmader et al., 2008; Inzlicht et al., 2011; Ståhl et al., 2012a).

### Conclusions Regarding Potential Hidden Costs of Coping With Stigma in the Workplace

In summary then, while we know individuals have various creative strategies available to cope with negative stereotypes, prejudice and discrimination, the regulation involved can take a significant toll. Members of negatively stereotyped groups face not only the usual workplace task demands but also juggle regulation of stigma with all its consequences. This regulation includes complex choices about whether to confront or not confront injustice and whether to display or hide one’s identity – staying with or distancing from the stigmatized group. Moreover, regulation strategies successful for the individual may have unintended negative consequences for other group members. Also, regulating threat may have its own consequences – including cognitive depletion and emotional exhaustion, potentially leading to less effective functioning over time. This can have serious consequences for the self, the organization, and the ironic reinforcement of the stereotypes that caused the initial depletion and exhaustion. Fatigue from daily management of such issues may lead members of negatively stereotyped groups to opt out: leaving contexts and domains in which they are stigmatized and entering domains where they face fewer such challenges (Crocker et al., 1998; Ryan et al., 2008; Stephens and Levine, 2011; Kossek et al., 2016). Crucially, these phenomena are unlikely to be recognized as responses to identity threat and may instead be seen as individual problems and “choices” (Ryan et al., 2008; Stephens and Levine, 2011). Also, even if each specific cost were to be small, they can build up and accumulate. Important future research directions thus include obtaining a much greater understanding of these cumulative costs of facing stigma (for examples see, Pascoe and Smart Richman, 2009; Kogan et al., 2015; Van Dijk and van Engen, 2019).

## Implications for Organizations

A threat, support, and hidden costs approach to targets’ responses to stigma helps us understand why current workplace diversity efforts that tend to focus on either “fixing the perpetrator” (e.g., anti-bias training) or “fixing the victim” (mentoring programs etc.) are not always successful in attracting and retaining members of stigmatized groups, and provides insights as to how we can more effectively reduce workplace inequality. A fixing the perpetrator or victim approach is much too simple of an understanding that ignores much of the complex human cognition and behavior through which in- and exclusion takes place. As reviewed here, processes of in- and exclusion include inadvertent automatic stereotypes and biases and subtle devaluations. These are harder to identity but potentially even more potent. A fixing the perpetrator or victim approach tends to look for sources within individuals rather than in the larger work environment or interaction between individuals. A threat, support, and hidden costs approach to targets’ responses to stigma helps us understand why members of negatively stereotyped groups may experience higher levels of stress, depletion, and burn out in organizations; may underperform or appear less committed or motivated; and may not always take available opportunities. These responses should be understood not as dysfunctional responses – or as inherent group differences – but as consequences of the regulation of identity threat in efforts to maintain multiple and sometimes conflicting goals for esteem, belonging, and achievement. This regulation can also entail important, but less obvious hidden costs. Extra vigilance for stigma may mean members of stigmatized groups recruit extra resources and perform well or even excellently in the short run. However, they may also show cognitive depletion and exhaustion over time. Similarly, moving up on the organizational ladder importantly benefits enhancement of self, but may leave members of negatively stereotyped groups as loners in predominantly outgroup organizations much in need of identity safety and ingroup support, or may lead them to distance themselves from other members of their group in an attempt to fit in, leaving the status quo unchanged and the benefits of diversity for organizations uncultivated.

Key in this focus on threats, coping, support, and hidden costs is also that this approach considers members of stigmatized groups not as passive recipients of negative stereotypes and bias, but as active individuals pursuing multiple goals for esteem, belonging, and achievement. This approach is thus part of a shift away from a perspective on members of majority groups as perpetrators and members of stigmatized groups as victims, to a social psychology of intergroup relations that examines the interacting role of the high-status dominant group and the low-status stigmatized group within the contexts in which these interactions occur (Ellemers and Barreto, 2015).

Based on the insights described in this paper, a number of specific implications for organizations arise. First, organizations can do more to create awareness: awareness of how sometimes very subtle identity threats occur in work contexts and in daily interactions through underrepresentation, stereotypes, and an emphasis on domains associated with the dominant group. This also includes an awareness of which supportive contextual factors can reduce threat, and the potential hidden costs of regulating negative stereotypes, prejudice, and discrimination. Such awareness is particularly important among employees who function as gatekeepers in evaluation, selection, and promotion functions and committees, and among people in leadership positions who strongly impact organizational norms, climates, and policies.

Creating awareness should be approached using good state-of-the-art methods, and it is vital for organizations to understand that offered diversity training programs – and diversity initiatives more generally – are not always consistent with the research state-of-the-art and may backfire or actually increase stereotypes (Kalev et al., 2006; Dobbin and Kalev, 2013, 2018; Kaiser et al., 2013; Roberson et al., 2013; Dover et al., 2014; Moss-Racusin et al., 2014; Brady et al., 2015; Kirby et al., 2015; Gündemir and Galinsky, 2017). This is especially the case when such programs emphasize group membership and stereotypical differences; focus on “fixing the faults” of members of stigmatized groups; or when employees who are members of groups currently overrepresented in the organization feel that these efforts find their group at fault or put their group at a disadvantage. Additionally, for programs to be effective, it is important that they provide insight into how potential threats often manifest themselves in subtle ways in daily workplace interactions. Increased awareness of what is actually important in order to address threats, support, and hidden costs for members of stigmatized groups then allows the tackling of the subtle barriers involved. These approaches are often quite different than what is currently common in the organization: for instance, an organizational diversity contact point where employees can notify someone when experiencing discrimination is not likely to pick up on (and hence address) subtle daily devaluation cues. Instead, counteracting such cues involves systematically scanning the workplace for cues in organizational materials, images, policies and advertisements, and in task and position assignments. Also, it means understanding how these cues and stereotypes become salient in the day-to-day workplace – e.g., in interactions between colleagues, and creating attention to this in the organization.

Second, organizations can create better structures and procedures that take into account this knowledge on threat, coping, support, and hidden costs. We know from much research that we cannot get rid of stereotypes easily, but we can set up recruitment, selection, evaluation, and promotion procedures in organizations such that there is less opportunity for stereotypes to affect outcomes. These structures and procedures go against individuals’ inclinations as busy and time-stressed human beings, leaving less opportunity for biases to impact decisions. The diversity literature has extensive guidelines on how to do this, including the monitoring and feedback of diversity progress (e.g., through the organization’s demographic statistics), and ensuring accountability for this progress; the use of more standardized and transparent recruitment, selection, and promotion procedures; and extra efforts to support networks, mentoring, and the availability of role models and supportive career planning for members of stigmatized groups (see e.g., Bias Interrupters for a comprehensive site monitoring and continually updating the best state-of-the-art on structures and procedures to increase diversity at work).

Finally, organizations should concentrate on creating “identity-safe” environments in which identities are not negatively viewed but positively valued – paying particular attention to what the current identity cues communicate regarding the safety of different identities in the organization. Organizations can make use of the increasing knowledge with regard to the impact of daily hassles and cues; the positive impact of identity affirmation; and work on reducing the various potential triggers of threats to increase workplace equality. Organizations can pay specific attention to the availability of outgroup and ingroup support – also through networks, role models, and people in authority within the organization. As part of this, checking for representation of stigmatized groups is important, addressing both numerical underrepresentation and organizational visibility, also at different levels of the organizational hierarchy. The presence of a critical mass in the organization is key (often around 30% in the case of gender), making the category much less relevant and reducing the salience of stereotypes. Indeed, studies show that critical mass protects workplace satisfaction and performance by decreasing identity concerns (Allmendinger and Hackman, 1995; Niemann and Dovidio, 1998; Inzlicht and Ben-Zeev, 2000, 2003; Sekaquaptewa and Thompson, 2002, 2003). However, increased representation is not always possible, especially in the case of true minorities (e.g., sexual minorities) and then reducing any negative salience of these identities and providing positive value to identity becomes even more important. Also, as noted, creating an identity-safe environment includes attention to members of the majority or dominant group, making sure members of dominant majority groups too are included and have their perspectives valued (see also Kaiser et al., 2013; Dover et al., 2016). Identity safety also involves scanning the workplace for an inadvertent focus on domains traditionally associated with some (but not other) groups, and checking the necessity of this emphasis in job descriptions, organizational communications, reward structures, and organizational culture (see also, Danbold and Huo, 2017; Danbold and Bendersky, 2018). For example, the same job or task can often be described in different ways, such that it is less focused on one group’s traditional qualities and therefore becomes attractive to employees from different groups. Consistent with this, describing STEM careers as more communal (i.e., stressing collaboration and apprentice or mentoring models rather than independence; stressing societal benefits) increases women’s positivity toward STEM careers while not harming men’s positivity (Diekman et al., 2011). Of course, these are solutions that do not challenge existing stereotypical views of who excels in which domain, and thus a long-term and broader solution involves the reduction of stereotypes through which certain domains and characteristics are automatically linked to specific groups (Eagly and Karau, 2002; Else-Quest et al., 2010).

## Conclusion

Our current understanding of social inequality and how targets cope has followed a history from a focus on members of dominant groups as perpetrators and members of stigmatized groups as passive victims, to a focus on members of stigmatized groups as active agents regulating identity threat. Today, there is a much better understanding of how targets are affected by and deal with workplace stereotypes, prejudice, and discrimination: we know that workplaces differ in the amount and kinds of social identity threat, and how these manifest themselves in increasingly subtle ways. Members of stigmatized groups cope with these threats in various ways; protecting their goals and their well-being, motivation, and performance. Support, particularly contextual support, can play an important role in mitigating threat and supporting self-regulation. Recent research also increasingly shows the costs of threat regulation: costs for individuals, for their ingroup, and for organizations. Together, these insights provide important starting points for how organizations can more effectively reduce workplace inequalities.

## Author Contributions

CL wrote a first set-up and draft of the paper. LM, JV, SG, NS and CJ made adjustments and wrote additions. All authors provided literature and several rounds of feedback on different versions of the manuscript, and approved it for publication.

### Conflict of Interest Statement

The authors declare that the research was conducted in the absence of any commercial or financial relationships that could be construed as a potential conflict of interest.

## Footnotes

^1^Ni and Huo’s results suggest that the demotivating effects of perceiving outgroup bias can outweigh negative effects of internalizing ingroup bias, and indeed we agree that both these processes can have negative effects.

## References

[ref1] Ahlqvist S. London B. Rosenthal L. (2013). Unstable identity compatibility: how gender rejection sensitivity undermines the success of women in science, technology, engineering, and mathematics fields. Psychol. Sci. 24, 1644–1652. 10.1177/0956797613476048 23818652

[ref2] Allmendinger J. Hackman J. R. (1995). The more, the better? A four-nation study of the inclusion of women in symphony orchestras. Soc. Forces 74, 423–460. 10.1093/sf/74.2.423

[ref3] Anisman-Razin M. Saguy T. (2016). Reactions to tokenism: the role of individual characteristics in shaping responses to token decisions. Eur. J. Soc. Psychol. 46, 716–731. 10.1002/ejsp.2215

[ref4] Archbold C. A. Hassell K. D. Stichman A. J. (2010). Comparing promotion aspirations among female and male police officers. Int. J. Police Sci. Manag. 12, 287–303. 10.1350/ijps.2010.12.2.175

[ref5] Aronson J. Quinn D. M. Spencer S. J. (1998). “Stereotype threat and the academic underperformance of minorities and women” in Prejudice: The target’s perspective. eds. Swim J. K. Stangor C. (San Diego, CA: Academic Press), 83–103.

[ref500] Avery D. R. McKay P. F. Wilson D. C. Tonidandel S. (2007). Unequal attendance: The relationships between race, organizational diversity cues, and absenteeism. Pers. Psychol. 60, 875–902. 10.1111/j.1744-6570.2007.00094.x, PMID: 18361629

[ref6] Avery D. R. McKay P. F. Wilson D. C. (2008). What are the odds? How demographic similarity affects the prevalence of perceived employment discrimination. J. Appl. Psychol. 93, 235–249. 10.1037/0021-9010.93.2.235, PMID: 18361629

[ref7] Avery D. R. Volpone S. D. Mckay P. F. King E. B. Wilson D. C. (2012). Is relational demography relative? How employment status influences effects of supervisor-subordinate demographic similarity. J. Bus. Psychol. 27, 83–98. 10.1007/s10869-011-9230-9

[ref8] Avery D. R. Volpone S. D. Stewart R. W. Luksyte A. Hernandez M. McKay P. F. (2013). Examining the draw of diversity: how diversity climate perceptions affect job-pursuit intentions. Hum. Resour. Manag. 52, 175–194. 10.1002/hrm.21524

[ref9] Bakouri M. Staerklé C. (2015). Coping with structural disadvantage: overcoming negative effects of perceived barriers through bonding identities. Br. J. Soc. Psychol. 54, 648–670. 10.1111/bjso.12102, PMID: 25683895

[ref10] Barreto M. (2014). “Experiencing and coping with social stigma” in APA handbook of personality and social psychology. Vol. 2, eds. Mikulincer M. Shaver P. R. Dovidio J. F. Simpson J. A. (Washington, DC, US: American Psychological Association), 473–506.

[ref11] Barreto M. Ellemers N. (2005). The perils of political correctness: men’s and women’s responses to old-fashioned and modern sexist views. Soc. Psychol. Q. 68, 75–88. 10.1177/019027250506800106

[ref12] Baruch-Feldman C. Brondolo E. Ben-Dayan D. Schwartz J. (2002). Sources of social support and burnout, job satisfaction, and productivity. J. Occup. Health Psychol. 7, 84–93. 10.1037/1076-8998.7.1.84 11827236

[ref13] Baumeister R. F. Bratslavsky E. Muraven M. Tice D. M. (1998). Ego depletion: is the active self a limited resource? J. Pers. Soc. Psychol. 74, 1252–1265. 10.1037/0022-3514.74.5.1252, PMID: 9599441

[ref14] Baysu G. Phalet K. Brown R. (2014). Relative group size and minority school success: the role of intergroup friendship and discrimination experiences. Br. J. Soc. Psychol. 53, 328–349. 10.1111/bjso.12035, PMID: 23672186

[ref15] Becker J. C. (2012). The system-stabilizing role of identity management strategies: social creativity can undermine collective action for social change. J. Pers. Soc. Psychol. 103, 647–662. 10.1037/a0029240, PMID: 22746675

[ref16] Becker J. C. Glick P. Ilic M. Bohner G. (2011). Damned if she does, damned if she doesn’t: consequences of accepting versus confronting patronizing help for the female target and male actor. Eur. J. Soc. Psychol. 41, 761–773. 10.1002/ejsp.823

[ref17] Becker J. C. Tausch N. (2014). When group memberships are negative: the concept, measurement, and behavioral implications of psychological disidentification. Self Identity 13, 294–321. 10.1080/15298868.2013.819991

[ref18] Becker J. C. Wright S. C. Lubensky M. E. Zhou S. (2013). Friend or ally whether cross-group contact undermines collective action depends on what advantaged group members say (or don’t say). Personal. Soc. Psychol. Bull. 39, 442–455. 10.1177/0146167213477155 23504760

[ref19] Becker J. C. Zawadzki M. J. Shields S. A. (2014). Confronting and reducing sexism: a call for research on intervention. J. Soc. Issues 70, 603–614. 10.1111/josi.12081

[ref20] Begeny C. T. Huo Y. J. (2017). When identity hurts: how positive intragroup experiences can yield negative mental health implications for ethnic and sexual minorities. Eur. J. Soc. Psychol. 47, 803–817. 10.1002/ejsp.2292

[ref21] Begeny C. T. Huo Y. J. (2018). Is it always good to feel valued? The psychological benefits and costs of higher perceived status in one’s ethnic minority group. Group Process. Intergroup Relat. 21, 193–213. 10.1177/1368430216656922

[ref22] Bell-Scriber M. J. (2008). Nursing education research: warming the nursing education climate for traditional-age learners who are male. Nurs. Educ. Perspect. 29, 143–150. PMID:. 18575237

[ref23] Biernat M. Vescio T. K. Green M. L. (1996). Selective self-stereotyping. J. Pers. Soc. Psychol. 71, 1194–1209. 10.1037/0022-3514.71.6.1194, PMID: 8979386

[ref24] Blascovich J. Mendes W. B. Hunter S. B. Lickel B. (2000). “Stigma, threat, and social interactions” in The social psychology of stigma. eds. Heatherton T. F. Kleck R. E. Hebl M. R. Hull J. G. (New York, NY, US: Guilford Press), 307–333.

[ref25] Blascovich J. Spencer S. J. Quinn D. Steele C. (2001). African Americans and high blood pressure: the role of stereotype threat. Psychol. Sci. 12, 225–229. 10.1111/1467-9280.00340 11437305

[ref26] Boen F. Vanbeselaere N. (2000). Responding to membership of a low-status group: the effects of stablility, permeability and individual ability. Group Process. Intergroup Relat. 3, 41–62. 10.1177/1368430200031003

[ref27] Bongiorno R. Bain P. G. David B. (2014). If you’re going to be a leader, at least act like it! Prejudice towards women who are tentative in leader roles. Br. J. Soc. Psychol. 53, 217–234. 10.1111/bjso.12032, PMID: 23509967

[ref28] Brady L. M. Kaiser C. R. Major B. Kirby T. A. (2015). It’s fair for us: diversity structures cause women to legitimize discrimination. J. Exp. Soc. Psychol. 57, 100–110. 10.1016/j.jesp.2014.11.010

[ref29] Branscombe N. R. Ellemers N. Spears R. Doosje B. (1999a). “The context and content of social identity threat” in Social identity: Context, commitment, content. eds. Ellemers N. Spears R. Doosje B. (Oxford, UK: Blackwell), 35–58.

[ref30] Branscombe N. R. Fernández S. Gómez A. Cronin T. (2012). “Moving toward or away from a group identity: different strategies for coping with pervasive discrimination” in The social cure: Identity, health and well-being. eds. Jetten J. Haslam S. A. (New York: Psychology Press), 115–131.

[ref31] Branscombe N. R. Schmitt M. T. Harvey R. D. (1999b). Perceiving pervasive discrimination among African Americans: implications for group identification and well-being. J. Pers. Soc. Psychol. 77, 135–149. 10.1037/0022-3514.77.1.135,

[ref32] Brown K. T. (2015). Perceiving allies from the perspective of non-dominant group members: comparisons to friends and activists. Curr. Psychol. 34, 713–722. 10.1007/s12144-014-9284-8

[ref33] Burkley A. Blanton H. (2008). Endorsing a negative in-group stereotype as a self-protective strategy: sacrificing the group to save the self. J. Exp. Soc. Psychol. 44, 37–49. 10.1016/j.jesp.2007.01.008

[ref34] Cadinu M. Maass A. Rosabianca A. Kiesner J. (2005). Why do women underperform under stereotype threat? Evidence for the role of negative thinking. Psychol. Sci. 16, 572–578. 10.1111/j.0956-7976.2005.01577.x, PMID: 16008792

[ref35] Carver C. S. Scheier M. F. (1998). On the self-regulation of behavior. Cambridge: Cambridge University Press.

[ref36] Carver C. S. Scheier M. F. (2002). Control processes and self-organization as complementary principles underlying behavior. Personal. Soc. Psychol. Rev. 6, 304–315. 10.1207/S15327957PSPR0604_05

[ref37] Cejka M. A. Eagly A. H. (1999). Gender stereotypic images of occupations correspond to the sex segregation of employment. Personal. Soc. Psychol. Bull. 25, 413–423. 10.1177/0146167299025004002

[ref38] Chaney K. E. Sanchez D. T. Remedios J. D. (2016). Organizational identity safety cue transfers. Personal. Soc. Psychol. Bull. 42, 1564–1576. 10.1177/0146167216665096, PMID: 30208783

[ref39] Cheryan S. Plaut V. C. Davies P. G. Steele C. M. (2009). Ambient belonging: how stereotypical cues impact gender participation in computer science. J. Pers. Soc. Psychol. 97, 1045–1060. 10.1037/a0016239, PMID: 19968418

[ref40] Cheryan S. Ziegler S. A. Montoya A. K. Jiang L. (2017). Why are some STEM fields more gender balanced than others? Psychol. Bull. 143, 1–35. 10.1037/bul0000052, PMID: 27732018

[ref41] Choi S. Rainey H. G. (2013). Organizational fairness and diversity management in public organizations: does fairness matter in managing diversity? Rev. Public Pers. Adm. 34, 307–331. 10.1177/0734371X13486489

[ref42] Cihangir S. Barreto M. Ellemers N. (2014). Men as allies against sexism: the positive effects of a suggestion of sexism by male (vs. female) sources. SAGE Open 4, 1–12. 10.1177/2158244014539168

[ref43] Cochran M. M. Brassard J. A. (1979). Child development and personal social networks. Child Dev. 50, 601–616. 10.2307/1128926

[ref44] Cohen G. L. Garcia J. (2005). “I Am Us”: negative stereotypes as collective threats. J. Pers. Soc. Psychol. 89, 566–582. 10.1037/0022-3514.89.4.566, PMID: 16287419

[ref45] Cohen L. L. Swim J. K. (1995). The differential impact of gender ratios on women and men: tokenism, self-confidence, and expectations. Personal. Soc. Psychol. Bull. 21, 876–884. 10.1177/0146167295219001

[ref46] Cohen S. Underwood L. G. Gottlieb B. H. (2000). Social support measurement and intervention. New York: Oxford University Press.

[ref47] Cohen S. Wills T. A. (1985). Stress, social support, and the buffering hypothesis. Psychol. Bull. 98, 310–357. 10.1037/0033-2909.98.2.310, PMID: 3901065

[ref48] Cole E. R. (2009). Intersectionality and research in psychology. Am. Psychol. 64, 170–180. 10.1037/a0014564, PMID: 19348518

[ref49] Compas B. E. Connor-Smith J. K. Saltzman H. Thomsen A. H. Wadsworth M. E. (2001). Coping with stress during childhood and adolescence: problems, progress, and potential in theory and research. Psychol. Bull. 127, 87–127. 10.1037/0033-2909.127.1.87, PMID: 11271757

[ref50] Connor-Smith J. K. Compas B. E. Wadsworth M. E. Thomsen A. H. Saltzman H. (2000). Responses to stress in adolescence: measurement of coping and involuntary stress responses. J. Consult. Clin. Psychol. 68, 976–992. 10.1037/0022-006X.68.6.976, PMID: 11142550

[ref51] Correll J. Park B. (2005). A model of the ingroup as a social resource. Personal. Soc. Psychol. Rev. 9, 341–359. 10.1207/s15327957pspr0904_4 16223356

[ref52] Cortina L. (2008). Unseen injustice: incivility as modern discrimination in organizations. Acad. Manag. Rev. 33, 55–75. 10.5465/amr.2008.27745097

[ref53] Crandall C. S. Merman A. Hebl M. (2009). “Anti-fat prejudice” in Handbook of prejudice, stereotyping, and discrimination. ed. Nelson T. D. (New York, NY: Psychology Press), 469–487.

[ref54] Creed W. E. D. (2006). “Seven conversations about the same thing: homophobia and heterosexism in the workplace” in Handbook of workplace diversity. eds. Konrad A. M. Prasad P. Pringle J. K. (Thousand Oaks, CA: Sage Publications), 371–400.

[ref55] Crisp R. J. Bache L. M. Maitner A. T. (2009). Dynamics of social comparison in counter-stereotypic domains: stereotype boost, not stereotype threat, for women engineering majors. Soc. Influ. 4, 171–184. 10.1080/15534510802607953

[ref56] Crocker J. Major B. (1989). Social stigma and self-esteem: the self-protective properties of stigma. Psychol. Rev. 96, 608–630. 10.1037/0033-295X.96.4.608

[ref57] Crocker J. Major B. Steele C. M. (1998). “Social stigma” in The handbook of social psychology. Vol. 4, eds. Gilbert D. T. Fiske S. T. Lindzey G. (Boston, MA: Mcgraw-Hill), 504–553.

[ref58] Croft A. Schmader T. Block K. (2015). An underexamined inequality: cultural and psychological barriers to men’s engagement with communal roles. Personal. Soc. Psychol. Rev. 19, 343–370. 10.1177/1088868314564789 25576312

[ref59] Crosby F. J. Cordova D. I. Jaskar K. (1993). “On the failure to see oneself as disadvantaged: cognitive and emotional components” in Group motivation: Social psychological perspectives. eds. Hogg M. A. Abrams D. (London, UK: Harvester Wheatsheaf), 87–104.

[ref60] Czopp A. M. Monteith M. J. (2003). Confronting prejudice (literally): reactions to confrontations of racial and gender bias. Personal. Soc. Psychol. Bull. 29, 532–544. 10.1177/0146167202250923, PMID: 15273006

[ref61] Czopp A. M. Monteith M. J. Mark A. Y. (2006). Standing up for a change: reducing bias through interpersonal confrontation. J. Pers. Soc. Psychol. 90, 784–803. 10.1037/0022-3514.90.5.784, PMID: 16737373

[ref62] Danbold F. Bendersky C. (2018). Inverting professional prototypes increases the valuation of women in male-dominated professions. Acad. Manag. Proc. 2018:17406. 10.5465/AMBPP.2018.200

[ref63] Danbold F. Huo Y. J. (2017). Men’s defense of their prototypicality undermines the success of women in STEM initiatives. J. Exp. Soc. Psychol. 72, 57–66. 10.1016/j.jesp.2016.12.014

[ref64] Dasgupta N. (2011). Ingroup experts and peers as social vaccines who inoculate the self-concept: the stereotype inoculation model. Psychol. Inq. 22, 231–246. 10.1080/1047840X.2011.607313

[ref65] Davies P. G. Spencer S. J. Steele C. M. (2005). Clearing the air: identity safety moderates the effects of stereotype threat on women’s leadership aspirations. J. Pers. Soc. Psychol. 88, 276–287. 10.1037/0022-3514.88.2.276 15841859

[ref66] De Lemus S. Bukowski M. Spears R. Telga M. (2015). Reactance to (or acceptance of) stereotypes: implicit and explicit responses to group identity threat. J. Psychol. 223, 236–246. 10.1027/2151-2604/a000225

[ref67] De Lemus S. Spears R. Bukowski M. Moya M. Lupiáñez J. (2013). Reversing implicit gender stereotype activation as a function of exposure to traditional gender roles. Soc. Psychol. 44, 109–116. 10.1027/1864-9335/a000140

[ref68] De Lemus S. Spears R. Van Breen J. A. Telga M. (2016). “Coping with identity threat: from implicit resistance to active control” in Coping with lack of control in a social world. eds. Bukowski M. Fritsche I. Guinote A. Kofta M. (London: Routledge), 151–169.

[ref69] Deaux K. Lafrance M. (1998). “Gender” in The handbook of social psychology. Vol. 2, eds. Gilbert D. Fiske S. Lindzey G. (New York, NY: McGraw-Hill), 788–827.

[ref70] Dennehy T. C. Dasgupta N. (2017). Female peer mentors early in college increase women’s positive academic experiences and retention in engineering. Proc. Natl. Acad. Sci. USA 114, 5964–5969. 10.1073/pnas.1613117114, PMID: 28533360 PMC5468611

[ref71] Derks B. Ellemers N. Van Laar C. de Groot K. (2011a). Do sexist organizational cultures create the queen bee? Br. J. Soc. Psychol. 50, 519–535. 10.1348/014466610X525280 21884548

[ref72] Derks B. Scheepers D. Van Laar C. Ellemers N. (2011b). The threat vs. the challenge of car parking for women: how self-group and group affirmation affect cardiovascular responses. J. Exp. Soc. Psychol. 47, 178–183. 10.1016/j.jesp.2010.08.016

[ref73] Derks B. Van Laar C. Ellemers N. (2006). Striving for success in outgroup settings: effects of contextually emphasizing ingroup dimensions on stigmatized group members’ social identity and performance styles. Personal. Soc. Psychol. Bull. 32, 576–588. 10.1177/0146167205283336 16702152

[ref74] Derks B. Van Laar C. Ellemers N. (2007a). Social creativity strikes back: improving motivated performance of low status group members by valuing ingroup dimensions. Eur. J. Soc. Psychol. 37, 490–493. 10.1002/ejsp.375

[ref75] Derks B. Van Laar C. Ellemers N. (2007b). The beneficial effects of social identity protection on the performance motivation of members of devalued groups. Soc. Issues Policy Rev. 1, 217–256. 10.1111/j.1751-2409.2007.00008.x

[ref76] Derks B. Van Laar C. Ellemers N. (2009). Working for the self or working for the group: how self- versus group affirmation affects collective behavior in low-status groups. J. Pers. Soc. Psychol. 96, 183–202. 10.1037/a0013068, PMID: 19210074

[ref77] Derks B. Van Laar C. Ellemers N. (2016). The queen bee phenomenon: why women leaders distance themselves from junior women. Leadersh. Q. 27, 456–469. 10.1016/j.leaqua.2015.12.007

[ref78] Derks B. Van Laar C. Ellemers N. de Groot K. (2011c). Gender-bias primes elicit queen-bee responses among senior policewomen. Psychol. Sci. 22, 1243–1249. 10.1177/0956797611417258 21873568

[ref79] Derks B. Van Laar C. Ellemers N. Raghoe G. (2015). Extending the queen bee effect: how Hindustani workers cope with disadvantage by distancing the self from the group. J. Soc. Issues 71, 476–496. 10.1111/josi.12124

[ref80] Diekman A. B. Clark E. K. Johnston A. M. Brown E. R. Steinberg M. (2011). Malleability in communal goals and beliefs influences attraction to stem careers: evidence for a goal congruity perspective. J. Pers. Soc. Psychol. 101, 902–918. 10.1037/a0025199, PMID: 21859224

[ref81] Diekman A. B. Hirnisey L. (2007). The effect of context on the silver ceiling: a role congruity perspective on prejudiced responses. Personal. Soc. Psychol. Bull. 33, 1353–1366. 10.1177/0146167207303019 17933733

[ref82] Dobbin F. Kalev A. (2013). “The origins and effects of corporate diversity programs” in Oxford handbook of diversity and work. ed. Roberson Q. M. (New York, NY: Oxford University Press), 253–281.

[ref83] Dobbin F. Kalev A. (2018). Why diversity training doesn’t work: the challenge for industry and academia. Anthropol. Now 10, 48–55. 10.1080/19428200.2018.1493182

[ref84] Dover T. Major B. Kaiser C. R. (2014). Diversity initiatives, status, and system-justifying beliefs: when and how diversity efforts de-legitimize discrimination claims. Group Process. Intergroup Relat. 17, 485–493. 10.1177/1368430213502560

[ref85] Dover T. Major B. Kaiser C. (2016). Members of high-status groups are threatened by pro-diversity organizational messages. J. Exp. Soc. Psychol. 62, 58–67. 10.1016/j.jesp.2015.10.006,

[ref86] Dovidio J. F. (2001). On the nature of contemporary prejudice: the third wave. J. Soc. Issues 57, 829–849. 10.1111/0022-4537.00244

[ref87] Dovidio J. F. Gaertner S. L. Kawakami K. (2010a). “Racism” in The SAGE handbook of prejudice, stereotyping, and discrimination. eds. Dovidio J. F. Hewstone M. Glick P. Esses V. M. (London, UK: Sage), 312–327.

[ref88] Dovidio J. F. Hewstone M. Glick P. Esses V. M. (2010b). “Prejudice, stereotyping and discrimination: theoretical and empirical overview” in The SAGE handbook of prejudice, stereotyping, and discrimination. eds. Dovidio J. F. Hewstone M. Glick P. Esses V. M. (London, UK: Sage), 3–28.

[ref89] Droogendyk L. Wright S. C. Lubensky M. Louis W. R. (2016). Acting in solidarity: cross-group contact between disadvantaged group members and advantaged group allies. J. Soc. Issues 72, 315–334. 10.1111/josi.12168

[ref90] Drury B. J. Kaiser C. R. (2014). Allies against sexism: the role of men in confronting sexism. J. Soc. Issues 70, 637–652. 10.1111/josi.12083,

[ref91] Duguid M. (2011). Female tokens in high-prestige work groups: catalysts or inhibitors of group diversification? Organ. Behav. Hum. Decis. Process. 116, 104–115. 10.1016/j.obhdp.2011.05.009

[ref92] Eagly A. H. (2016). When passionate advocates meet research on diversity, does the honest broker stand a chance? J. Soc. Issues 72, 199–222. 10.1111/josi.12163

[ref93] Eagly A. H. Karau S. J. (2002). Role congruity theory of prejudice toward female leaders. Psychol. Rev. 109, 573–598. 10.1037/0033-295X.109.3.573, PMID: 12088246

[ref94] Eccles J. S. (1994). Understanding women’s educational and occupational choices: applying the Eccles et al. model of achievement-related choices. Psychol. Women Q. 18, 585–609. 10.1111/j.1471-6402.1994.tb01049.x

[ref95] Eguchi S. (2009). Negotiating hegemonic masculinity: the rhetorical strategy of “straight-acting” among gay men. J. Intercult. Commun. Res. 38, 193–209. 10.1080/17475759.2009.508892

[ref96] Eliezer D. Major B. (2012). It’s not your fault: the social costs of claiming discrimination on behalf of someone else. Group Process. Intergroup Relat. 15, 487–502. 10.1177/1368430211432894

[ref97] Ellemers N. Barreto M. (2015). Modern discrimination: how perpetrators and targets interactively perpetuate social disadvantage. Curr. Opin. Behav. Sci. 3, 142–146. 10.1016/j.cobeha.2015.04.001

[ref98] Ellemers N. Rink F. (2016). Diversity in work groups. Curr. Opin. Psychol. 11, 49–53. 10.1016/j.copsyc.2016.06.001

[ref99] Ellemers N. Spears R. Doosje B. (1999). Social identity: Context, commitment, content. Oxford, UK: Blackwell.

[ref100] Ellemers N. Spears R. Doosje B. (2002). Self and social identity. Annu. Rev. Psychol. 53, 161–186. 10.1146/annurev.psych.53.100901.135228 11752483

[ref101] Ellemers N. van den Heuvel H. de Gilder D. Maass A. Bonvini A. (2004). The underrepresentation of women in science: differential commitment or the queen bee syndrome? Br. J. Soc. Psychol. 43, 315–338. 10.1348/0144666042037999, PMID: 15479533

[ref102] Ellemers N. Van Laar C. (2010). “Individual mobility” in The SAGE handbook of prejudice, stereotyping, and discrimination. eds. Dovidio J. Hewstone M. Glick P. Esses V. (London, UK: Sage), 561–576.

[ref103] Else-Quest N. M. Hyde J. S. Linn M. C. (2010). Cross-national patterns of gender differences in mathematics: a meta-analysis. Psychol. Bull. 136, 103–127. 10.1037/a0018053, PMID: 20063928

[ref104] Emerson K. T. U. Murphy M. C. (2014). Identity threat at work: how social identity threat and situational cues contribute to racial and ethnic disparities in the workplace. Cult. Divers. Ethn. Minor. Psychol. 20, 508–520. 10.1037/a0035403, PMID: 25133411

[ref105] Emina S. Hardacre S. Elton B. Branscombe N. R. Ryan M. K. Reynolds K. J. (2018). “We for She”: mobilising men and women to act in solidarity for gender equality. Group Process. Intergroup Relat. 21, 707–724. 10.1177/1368430218763272

[ref106] Faniko K. Ellemers N. Derks B. Lorenzi-Cioldi F. (2017). Nothing changes, really: why women who break through the glass ceiling end up reinforcing it. Personal. Soc. Psychol. Bull. 43, 638–651. 10.1177/0146167217695551, PMID: 28903635 PMC5414903

[ref107] Fingerhut A. W. (2011). Straight allies: what predicts heterosexuals’ alliance with the LGBT community? J. Appl. Soc. Psychol. 41, 2230–2248. 10.1111/j.1559-1816.2011.00807.x

[ref108] Fiske S. T. (2004). Social Beings: A core motives approach to social psychology. New York: Wiley.

[ref109] Fiske S. T. (2008). “Core social motivations: views from the couch, consciousness, classroom, computers, and collectives” in Handbook of motivation science. eds. Shah J. Y. Gardner W. L. (New York, NY, US: Guilford Press), 3–22.

[ref110] Fiske S. T. Bersoff D. N. Borgida E. Deaux K. Heilman M. E. (1991). Social science research on trial: use of sex stereotyping research in Price Waterhouse v. Hopkins. Am. Psychol. 46, 1049–1060. 10.1037/0003-066X.46.10.1049

[ref111] Fiske S. T. Cuddy A. J. C. Glick P. Xu J. (2002). A model of (often mixed) stereotype content: competence and warmth respectively follow from perceived status and competition. J. Pers. Soc. Psychol. 82, 878–902. 10.1037/0022-3514.82.6.878, PMID: 12051578

[ref112] Frable D. E. S. Platt L. Hoey S. (1998). Concealable stigmas and positive self-perceptions: feeling better around similar others. J. Pers. Soc. Psychol. 74, 909–922. 10.1037/0022-3514.74.4.909, PMID: 9569651

[ref113] Frey F. E. Tropp L. R. (2006). Being seen as individuals versus as group members: extending research on metaperception to intergroup contexts. Personal. Soc. Psychol. Rev. 10, 265–280. 10.1207/s15327957pspr1003_5 16859441

[ref114] Gabriel A. S. Butts M. M. Yuan Z. Rosen R. L. Sliter M. T. (2017). Further understanding incivility in the workplace: the effects of gender, agency, and communion. J. Appl. Psychol. 103, 362–382. 10.1037/apl0000289 29239641

[ref115] Garcia J. A. Crocker J. (2008). Reasons for disclosing depression matter: the consequences of having egosystem and ecosystem goals. Soc. Sci. Med. 67, 453–462. 10.1016/j.socscimed.2008.03.016, PMID: 18450349

[ref116] Gaucher D. Friesen J. Kay A. C. (2011). Evidence that gendered wording in job advertisements exists and sustains gender inequality. J. Pers. Soc. Psychol. 101, 109–128. 10.1037/a0022530, PMID: 21381851

[ref117] Gervais S. J. Hillard A. L. (2014). Confronting sexism as persuasion: effects of a confrontation’s recipient, source, message, and context. J. Soc. Issues 70, 653–667. 10.1111/josi.12084

[ref118] Ghavami N. Fingerhut A. Peplau L. A. Grant S. K. Wittig M. A. (2011). Testing a model of minority identity achievement, identity affirmation, and psychological well-being among ethnic minority and sexual minority individuals. Cultur. Divers. Ethnic Minor. Psychol. 17, 79–88. 10.1037/a0022532, PMID: 21341900 PMC3158128

[ref119] Goldman B. M. Kernis M. H. (2002). The role of authenticity in healthy psychological functioning and subjective well-being. Ann. Am. Psychother. Assoc. 5, 18–20.

[ref120] Gonzalez J. A. Denisi A. S. (2009). Cross-level effects of demography and diversity climate on organizational attachment and firm effectiveness. J. Organ. Behav. 30, 21–40. 10.1002/job.498

[ref121] Good J. J. Sanchez D. T. Moss-Racusin C. A. (2018). A paternalistic duty to protect? Predicting men’s decisions to confront sexism. Psychol. Men Masculinity 19, 14–24. 10.1037/men0000077

[ref122] Grimm L. R. Markman A. B. Maddox W. T. Baldwin G. C. (2009). Stereotype threat reinterpreted as a regulatory mismatch. J. Pers. Soc. Psychol. 96, 288–304. 10.1037/a0013463, PMID: 19159133 PMC2630514

[ref123] Gündemir S. Galinsky A. D. (2017). Multicolored blindfolds: how organizational multiculturalism can conceal racial discrimination and delegitimize racial discrimination claims. Soc. Psychol. Personal. Sci. 9, 825–834. 10.1177/1948550617726830

[ref124] Gündemir S. Homan A. C. Usova A. Galinsky A. D. (2017). Multicultural meritocracy: the synergistic benefits of valuing diversity and merit. J. Exp. Soc. Psychol. 73, 34–41. 10.1016/j.jesp.2017.06.002

[ref125] Hall W. Schmader T. Aday A. Croft E. (2019). Decoding the dynamics of social identity threat in the workplace: a within-person analysis of women’ s and men’s interactions in STEM. Soc. Psychol. Personal. Sci. 10, 542–552. 10.1177/1948550618772582

[ref126] Hall W. Schmader T. Aday A. Inness M. Croft E. (2018). Climate control: the relationship between social identity threat and cues to an identity-safe culture. J. Pers. Soc. Psychol. 115, 446–467. 10.1037/pspi0000137, PMID: 30047760

[ref127] Hall W. M. Schmader T. Croft A. (2015). Engineering exchanges: daily social identity threat predicts burnout among female engineers. Soc. Psychol. Personal. Sci. 6, 528–534. 10.1177/1948550615572637

[ref128] Hartman H. Hartman M. (2008). How undergraduate engineering students perceive women’s (and men’s) problems in science, math and engineering. Sex Roles 58, 251–265. 10.1007/s11199-007-9327-9

[ref129] Haslam S. A. O’Brien A. Jetten J. Vormedal K. Penna S. (2005). Taking the strain: social identity, social support, and the experience of stress. Br. J. Soc. Psychol. 44, 355–370. 10.1348/014466605X37468, PMID: 16238844

[ref130] Hebl M. R. Foster J. B. Mannix L. M. Dovidio J. F. (2002). Formal and interpersonal discrimination: a field study of bias toward homosexual applicants. Personal. Soc. Psychol. Bull. 28, 815–825. 10.1177/0146167202289010

[ref131] Hebl M. R. M. Law C. L. King E. (2010). “Heterosexism” in The SAGE handbook of prejudice, stereotyping and discrimination. eds. Dovidio J. F. Hewstone M. Glick P. Esses V. M. London, UK: Sage 345–360.

[ref132] Heilman M. E. (1983). “Sex bias in work settings: the lack of fit model” in Research in organizational behavior. Vol. 5, eds. Staw B. Cummings L., (Greenwich, CT: JAI Press), 269–298.

[ref133] Heilman M. E. (2012). Gender stereotypes and workplace bias. Res. Organ. Behav. 32, 113–135. 10.1016/j.riob.2012.11.003

[ref134] Hentschel T. Shemla M. Wegge J. Kearney E. (2013). Perceived diversity and team functioning: the role of diversity beliefs and affect. Small Group Res. 44, 33–61. 10.1177/1046496412470725

[ref135] Herek G. M. (2009). “Sexual prejudice” in Handbook of prejudice, stereotyping, and discrimination. ed. Nelson T. D. (New York, NY, US: Psychology Press), 441–467.

[ref136] Hersby M. D. Ryan M. K. Jetten J. (2009). Getting together to get ahead: the impact of social structure on women’s networking. Br. J. Manag. 20, 415–430. 10.1111/j.1467-8551.2008.00604.x

[ref137] Higgins E. T. (2012). Beyond pleasure and pain: how motivation works. Am. Psychol. 52, 1280–1300. 10.1093/acprof:oso/9780199765829.001.0001 9414606

[ref138] Hornsey M. J. Imani A. (2004). Criticizing groups from the inside and the outside: an identity perspective on the intergroup sensitivity effect. Personal. Soc. Psychol. Bull. 30, 365–383. 10.1177/0146167203261295 15030626

[ref139] Hornsey M. J. Oppes T. Svensson A. (2002). “It’s OK if we say it, but you can’t”: responses to intergroup and intragroup criticism. Eur. J. Soc. Psychol. 32, 293–307. 10.1002/ejsp.90

[ref140] House J. S. Umberson D. Landis K. R. (1988). Structures and processes of social support. Annu. Rev. Sociol. 14, 293–318. 10.1146/annurev.so.14.080188.001453

[ref141] Hoyt C. L. Blascovich J. (2007). Leadership efficacy and women leaders’ responses to stereotype activation. Group Process. Intergroup Relat. 10, 595–616. 10.1177/1368430207084718

[ref142] Hoyt C. L. Blascovich J. (2010). The role of leadership self-efficacy and stereotype activation on cardiovascular, behavioral and self-report responses in the leadership domain. Leadersh. Q. 21, 89–103. 10.1016/j.leaqua.2009.10.007

[ref143] Hunt C. J. Fasoli F. Carnaghi A. Cadinu M. (2016). Masculine self-presentation and distancing from femininity in gay men: an experimental examination of the role of masculinity threat. Psychol. Men Masculinity 17, 108–112. 10.1037/a0039545

[ref144] Huo Y. J. Molina L. E. (2006). Is pluralism a viable model of diversity? The benefits and limits of subgroup respect. Group Process. Intergroup Relat. 9, 359–376. 10.1177/1368430206064639

[ref145] Inzlicht M. Ben-Zeev T. (2000). A threatening intellectual environment: why females are susceptible to experiencing problem-solving deficits in the presence of males. Psychol. Sci. 11, 365–371. 10.1111/1467-9280.00272 11228906

[ref146] Inzlicht M. Ben-Zeev T. (2003). Do high-achieving female students underperform in private? The implications of threatening environments on intellectual processing. J. Educ. Psychol. 95, 796–805. 10.1037/0022-0663.95.4.796

[ref147] Inzlicht M. Legault L. (2014). “No pain, no gain: how distress underlies effective self-control (and unites diverse social psychological phenomena)” in Sydney symposium of social psychology. Motivation and its regulation: The control within. eds. Forgas J. P. Harmon-Jones E. (New York, NY: Psychology Press), 115–132.

[ref148] Inzlicht M. Schmader T. (2012). Stereotype threat: Theory, process, and application. New York, NY: Oxford University Press.

[ref149] Inzlicht M. Tullett A. M. Legault L. Kang S. K. (2011). Lingering effects: stereotype threat hurts more than you think. Soc. Issues Policy Rev. 5, 227–256. 10.1111/j.1751-2409.2011.01031.x

[ref150] Iyer A. Ryan M. K. (2009). Why do men and women challenge gender discrimination in the workplace? The role of group status and in-group identification in predicting pathways to collective action. J. Soc. Issues 65, 791–814. 10.1111/j.1540-4560.2009.01625.x

[ref151] Jackson P. Thoits P. Taylor H. (1995). Composition of the workplace and psychological well-being: the effects of tokenism on America’s black elite. Soc. Forces 74, 543–557. 10.1093/sf/74.2.543

[ref152] Jetten J. Haslam C. Haslam S. A. Dingle G. Jones J. M. (2014). How groups affect our health and well-being: the path from theory to policy. Soc. Issues Policy Rev. 8, 103–130. 10.1111/sipr.12003

[ref153] Johns M. Inzlicht M. Schmader T. (2008). Stereotype threat and executive resource depletion: examining the influence of emotion regulation. J. Exp. Psychol. Gen. 137, 691–705. 10.1037/a0013834 18999361 PMC2976617

[ref154] Johnson S. E. Mitchell M. A. Bean M. G. Richeson J. A. Shelton J. N. (2010). Gender moderates the self-regulatory consequences of suppressing emotional reactions to sexism. Group Process. Intergroup Relat. 13, 215–226. 10.1177/1368430209344867

[ref155] Jones K. P. Peddie C. I. Gilrane V. L. King E. B. Gray A. L. (2016). Not so subtle: a meta-analytic investigation of the correlates of subtle and overt discrimination. J. Manag. 42, 1588–1613. 10.1177/0149206313506466

[ref156] Kaiser C. R. (2006). “Dominant ideology threat and the interpersonal consequences of attributions to discrimination” in Stigma and group inequality: Social psychological approaches. eds. Levin S. Van Laar C. (Mahwah, NJ: Lawrence Erlbaum), 45–64.

[ref157] Kaiser C. R. Major B. Jurcevic I. Dover T. L. Brady L. M. Shapiro J. R. (2013). Presumed fair: ironic effects of organizational diversity structures. J. Pers. Soc. Psychol. 104, 504–519. 10.1037/a0030838, PMID: 23163748

[ref158] Kaiser C. R. Miller C. T. (2003). Derogating the victim: the interpersonal consequences of blaming events on discrimination. Group Process. Intergroup Relat. 6, 227–237. 10.1177/13684302030063001

[ref159] Kaiser C. R. Spalding K. E. (2015). Do women who succeed in male-dominated domains help other women? The moderating role of gender identification. Eur. J. Soc. Psychol. 45, 599–608. 10.1002/ejsp.2113

[ref160] Kaiser C. R. Vick S. B. Major B. (2006). Prejudice expectations moderate preconscious attention to cues that are threatening to social identity. Psychol. Sci. 17, 332–338. 10.1111/j.1467-9280.2006.01707.x 16623691

[ref161] Kalev A. Dobbin F. Kelly E. (2006). Best practices or best guesses? Assessing the efficacy of corporate affirmative action and diversity policies. Am. Sociol. Rev. 71, 589–617. 10.1177/000312240607100404

[ref162] Kalokerinos E. Kjelsaas K. Bennetts S. Hippel C. (2017). Men in pink collars: stereotype threat and disengagement among male teachers and child protection workers. Eur. J. Soc. Psychol. 47, 553–565. 10.1002/ejsp.2246,

[ref163] Kalokerinos E. K. von Hippel C. Zacher H. (2014). Is stereotype threat a useful construct for organizational psychology research and practice? Ind. Organ. Psychol. 7, 381–402. 10.1111/iops.12167

[ref164] Keller J. (2007). When negative stereotypic expectancies turn into challenge or threat: the moderating role of regulatory focus. Swiss J. Psychol. 66, 163–168. 10.1024/1421-0185.66.3.163

[ref165] Kirby T. A. Kaiser C. R. Major B. (2015). Insidious procedures: diversity awards legitimize unfair organizational practices. Soc. Justice Res 28, 169–186. 10.1007/s11211-015-0240-z

[ref166] Koch S. Holland R. W. Hengstler M. Van Knippenberg A. (2009). Body locomotion as regulatory process: stepping backward enhances cognitive control. Psychol. Sci. 20, 549–550. 10.1111/j.1467-9280.2009.02342.x, PMID: 19476588

[ref167] Koch S. Holland R. W. Van Knippenberg A. (2008). Regulating cognitive control through approach-avoidance motor actions. Cognition 109, 133–142. 10.1016/j.cognition.2008.07.014, PMID: 18835601

[ref168] Koenig A. M. Eagly A. H. (2014). Extending role congruity theory of prejudice to men and women with sex-typed mental illnesses. Basic Appl. Soc. Psychol. 36, 70–82. 10.1080/01973533.2013.856789

[ref169] Kogan S. M. Yu T. Allen K. A. Brody G. H. (2015). Racial microstressors, racial self-concept, and depressive symptoms among male African Americans during the transition to adulthood. J. Youth Adolesc. 44, 898–909. 10.1007/s10964-014-0199-3, PMID: 25344920 PMC4391463

[ref170] Kossek E. E. Su R. Wu L. (2016). “Opting out” or “pushed out”? Integrating perspectives on womens career equality for gender inclusion and interventions. J. Manag. 43, 1–27. 10.1177/0149206316671582

[ref171] Latrofa M. Vaes J. Cadinu M. (2012). Self-stereotyping: the central role of an ingroup threatening identity. J. Soc. Psychol. 152, 92–111. 10.1080/00224545.2011.565382, PMID: 22308763

[ref172] Lazarus R. S. Folkman S. (1984a). “Coping and adaptation” in The Handbook of behavioral medicine. ed. Gentry W. D. (New York: Guilford), 282–325.

[ref173] Lazarus R. S. Folkman S. (1984b). Stress, appraisal, and coping. New York, NY: Springer.

[ref174] Leach C. W. Livingston A. G. (2015). Contesting the meaning of inter-group disadvantage: towards a psychology of resistance. J. Soc. Issues 71, 614–632. 10.1111/josi.12131

[ref175] Leicht C. Goclowska M. A. Van Breen J. A. de Lemus S. de Moura G. R. (2017). Counter-stereotypes and feminism promote leadership aspirations in highly identified women. Front. Psychol. 8:883. 10.3389/fpsyg.2017.00883 28626437 PMC5454072

[ref176] Levin S. Van Laar C. Foote W. (2006). Ethnic segregation and perceived discrimination in college: mutual influences and effects on social and academic life. J. Appl. Soc. Psychol. 36, 1471–1501. 10.1111/j.0021-9029.2006.00068.x

[ref177] Liao H. Joshi A. Chuang A. (2004). Sticking out like a sore thumb: employee dissimilarity and deviance at work. Pers. Psychol. 57, 969–1000. 10.1111/j.1744-6570.2004.00012.x

[ref178] Lockwood P. (2006). “Someone like me can be successful”: do college students need same-gender role models? Psychol. Women Q. 30, 36–46. 10.1111/j.1471-6402.2006.00260.x

[ref179] Lockwood P. Kunda Z. (1999). Increasing the salience of one’s best selves can undermine inspiration by outstanding role models. J. Pers. Soc. Psychol. 76, 214–228. 10.1037/0022-3514.76.2.214, PMID: 10074706

[ref180] Logel C. Iserman E. C. Davies P. G. Quinn D. M. Spencer S. J. (2009). The perils of double consciousness: the role of thought suppression in stereotype threat. J. Exp. Soc. Psychol. 45, 299–312. 10.1016/j.jesp.2008.07.016

[ref181] London B. Rosenthal L. Gonzalez A. (2011a). Assessing the role of gender rejection sensitivity, identity, and support on the academic engagement of women in nontraditional fields using experience sampling methods. J. Soc. Issues 67, 510–530. 10.1111/j.1540-4560.2011.01712.x

[ref182] London B. Rosenthal L. Levy S. R. Lobel M. (2011b). The influences of perceived identity compatibility and social support on women in nontraditional fields during the college transition. Basic Appl. Soc. Psychol. 33, 304–321. 10.1080/01973533.2011.614166

[ref183] Luijters K. van der Zee K. I. Otten S. (2008). Cultural diversity in organizations: enhancing identification by valuing differences. Int. J. Intercult. Relat. 32, 154–163. 10.1016/j.ijintrel.2007.09.003

[ref184] Lyness K. S. Heilman M. E. (2006). When fit is fundamental: performance evaluations and promotions of upper-level female and male managers. J. Appl. Psychol. 91, 777–785. 10.1037/0021-9010.91.4.777, PMID: 16834505

[ref185] Major B. O’Brien L. T. (2005). The social psychology of stigma. Annu. Rev. Psychol. 56, 393–421. 10.1146/annurev.psych.56.091103.070137, PMID: 15709941

[ref186] Marques J. Paez D. (1994). The ‘black sheep effect’: social categorization, rejection of ingroup deviates, and perception of group variability. Eur. Rev. Soc. Psychol. 5, 37–68. 10.1080/14792779543000011

[ref187] Marx D. M. Ko S. J. Friedman R. A. (2009). The “Obama effect”: how a salient role model reduces race-based performance differences. J. Exp. Soc. Psychol. 45, 953–956. 10.1016/j.jesp.2009.03.012

[ref188] Marx D. M. Roman J. S. (2002). Female role models: protecting women’s math test performance. Personal. Soc. Psychol. Bull. 28, 1183–1193. 10.1177/01461672022812004

[ref501] McIntyre R. B. Lord C. G. Gresky D. M. Ten Eyck L. L. Frye G. D. J. Bond C. F. (2005). A social impact trend in the effects of role models on alleviating women’s mathematics stereotype threat. Curr. Res. Soc. Psychol. 10, 116–136.

[ref189] McIntyre R. B. Paulson R. M. Lord C. G. (2003). Alleviating women’s mathematics stereotype threat through salience of group achievements. J. Exp. Soc. Psychol. 39, 83–90. 10.1016/S0022-1031(02)00513-9

[ref190] McLeroy K. R. Gottlieb N. H. Heaney C. A. (2001). “Social health” in Health promotion in the workplace. 3rd Edn. eds. O’Donnell M. P. Harris J. S. (Albany, NY: Delmar).

[ref191] Meeussen L. Otten S. Phalet K. (2014). Managing diversity: how leaders’ multiculturalism and colorblindness affect work group functioning. Group Process. Intergroup Relat. 17, 629–644. 10.1177/1368430214525809

[ref192] Meeussen L. Van Laar C. Van Grootel S. (2019). How to foster male engagement tin traditionally female communal roles and occupations: Insights from research on gender norms and precarious manhood. To appear in: Social Issues and Policy Review.

[ref193] Mendes W. B. Jamieson J. (2012). “Embodied stereotype threat: exploring brain and body mechanisms underlying performance impairment” in Stereotype threat: Theory, process, and application. eds. Inzlicht M. Schmader T. (New York, NY: Oxford University Press), 51–68.

[ref194] Miller C. T. (2006). “Social psychological perspectives on coping with stressors related to stigma” in Stigma and group inequality: Social psychological approaches. eds. Levin S. Van Laar C. (Mahwah: Lawrence Erlbaum Associates Publishers), 21–44.

[ref195] Miller C. T. Kaiser C. R. (2001). A theoretical perspective on coping with stigma. J. Soc. Issues 57, 73–92. 10.1111/0022-4537.00202

[ref196] Miller C. T. Major B. (2000). “Coping with stigma and prejudice” in The social psychology of stigma. eds. Heatherton T. F. Kleck R. E. Hebl M. R. Hull J. G. (New York, NY, US: The Guilford Press), 243–272.

[ref197] Morgenroth T. Ryan M. K. Peters K. (2015). The motivational theory of role modeling: how role models influence role aspirants’ goals. Rev. Gen. Psychol. 19, 465–483. 10.1037/gpr0000059

[ref198] Moss-Racusin C. A. Van Der Toorn J. Dovidio J. F. Brescoll V. L. Graham M. J. Handelsman J. (2014). Scientific diversity interventions. Science 343, 615–616. 10.1126/science.1245936 24503840

[ref199] Muraven M. Baumeister R. F. (2000). Self-regulation and depletion of limited resources: does self-control resemble a muscle? Psychol. Bull. 126, 247–259. 10.1037/0033-2909.126.2.247, PMID: 10748642

[ref200] Murphy M. C. Steele C. M. Gross J. J. (2007). Signaling threat: how situational cues affect women in math, science, and engineering settings. Psychol. Sci. 18, 879–885. 10.1111/j.1467-9280.2007.01995.x 17894605

[ref201] Murphy M. C. Taylor V. J. (2012). “The role of situational cues in signaling and maintaining stereotype threat” in Stereotype threat: Theory, process, and application. eds. Inzlicht M. Schmader T. (New York, NY, US: Oxford University Press), 17–33.

[ref202] Nelson T. D. (2009). Handbook of prejudice, stereotyping, and discrimination. New York, NY: Psychology Press.

[ref203] Newheiser A.-K. Barreto M. (2014). Hidden costs of hiding stigma: ironic interpersonal consequences of concealing a stigmatized identity in social interactions. J. Exp. Soc. Psychol. 52, 58–70. 10.1016/j.jesp.2014.01.002

[ref204] Newheiser A.-K. Barreto M. Tiemersma J. (2017). People like me don’t belong here: identity concealment is associated with negative workplace experiences. J. Soc. Issues 73, 341–358. 10.1111/josi.12220

[ref205] Ni H. W. Huo Y. J. (2018). Same-gender supervisors protect women’s leadership aspirations after negative performance feedback. J. Appl. Soc. Psychol. 48, 437–447. 10.1111/jasp.12523

[ref206] Niemann Y. F. Dovidio J. F. (1998). Relationship of solo status, academic rank, and perceived distinctiveness to job satisfaction of racial/ethnic minorities. J. Appl. Psychol. 83, 55–71. 10.1037/0021-9010.83.1.55, PMID: 9494440

[ref207] Nishii L. H. (2013). The benefits of climate for inclusion for gender-diverse groups. Acad. Manag. J. 56, 1754–1774. 10.5465/amj.2009.0823

[ref208] O’Brien L. T. Blodorn A. Adams G. Garcia D. M. Hammer E. (2015). Ethnic variation in gender-STEM stereotypes and STEM participation: an intersectional approach. Cult. Divers. Ethn. Minor. Psychol. 21, 169–180. 10.1037/a0037944, PMID: 25244590

[ref209] O’Brien L. T. O. Hitti A. Shaffer E. Van Camp A. R. Henry D. Gilbert P. N. (2016). Improving girls sense of fit in science: increasing the impact of role models. Soc. Psychol. Personal. Sci. 8, 301–309. 10.1177/1948550616671997

[ref210] Öhman A. Mineka S. (2001). Fears, phobias, and preparedness: toward an evolved module of fear and fear learning. Psychol. Rev. 108, 483–522. 10.1037//0033-295X.108.3.483 11488376

[ref211] Operario D. Fiske S. T. (2001). Ethnic identity moderates perceptions of prejudice: judgments of personal versus group discrimination and subtle versus blatant bias. Personal. Soc. Psychol. Bull. 27, 550–561. 10.1177/0146167201275004

[ref212] Ostberg V. Lennartsson C. (2007). Getting by with a little help: the importance of various types of social support for health problems. Scand. J. Public Health 35, 197–204. 10.1080/14034940600813032 17454924

[ref213] Oswald D. L. Chapleau K. M. (2010). Selective self-stereotyping and women’s self-esteem maintenance. Personal. Individ. Differ. 49, 918–922. 10.1016/j.paid.2010.07.030

[ref214] Oyserman D. Swim J. K. (2001). Stigma: an insider’s view. J. Soc. Issues 57, 1–14. 10.1111/0022-4537.00198

[ref215] Pascoe E. A. Smart Richman L. S. (2009). Perceived discrimination and health: a meta-analytic review. Psychol. Bull. 135, 531–554. 10.1037/a0016059, PMID: 19586161 PMC2747726

[ref216] Pennington C. R. Heim D. Levy A. R. Larkin D. T. (2016). Twenty years of stereotype threat research: a review of psychological mediators. PLoS One 11:e0146487. 10.1371/journal.pone.0146487, PMID: 26752551 PMC4713435

[ref217] Plaut V. C. (2010). Diversity science: who needs it? Psychol. Inq. 21, 168–174. 10.1080/1047840X.2010.492753

[ref218] Plaut V. C. Garnett F. G. Buffardi L. E. Sanchez-Burks J. (2011). “What about me?” Perceptions of exclusion and Whites’ reactions to multiculturalism. J. Pers. Soc. Psychol. 101, 337–353. 10.1037/a0022832, PMID: 21534702

[ref219] Plaut V. C. Thomas K. M. Goren M. J. (2009). Is multiculturalism or color blindness better for minorities? Psychol. Sci. 20, 444–446. 10.1111/j.1467-9280.2009.02318.x 19399972

[ref220] Podsiadlowski A. Gröschke D. Kogler M. Springer C. Van Der Zee K. (2013). Managing a culturally diverse workforce: diversity perspectives in organizations. Int. J. Intercult. Relat. 37, 159–175. 10.1016/j.ijintrel.2012.09.001

[ref221] Pronin E. Steele C. M. Ross L. (2004). Identity bifurcation in response to stereotype threat: women and mathematics. J. Exp. Soc. Psychol. 40, 152–168. 10.1016/S0022-1031(03)00088-X

[ref222] Purdie-Vaughns V. Eibach R. P. (2008). Intersectional invisibility: the distinctive advantages and disadvantages of multiple subordinate-group identities. Sex Roles 59, 377–391. 10.1007/s11199-008-9424-4

[ref223] Purdie-Vaughns V. Steele C. M. Davies P. G. Ditlmann R. Crosby J. R. (2008). Social identity contingencies: how diversity cues signal threat or safety for African Americans in mainstream institutions. J. Pers. Soc. Psychol. 94, 615–630. 10.1037/0022-3514.94.4.615, PMID: 18361675

[ref224] Putman P. Roelofs K. (2011). Effects of single cortisol administrations on human affect reviewed: coping with stress through adaptive regulation of automatic cognitive processing. Psychoneuroendocrinology 36, 439–448. 10.1016/j.psyneuen.2010.12.001, PMID: 21194844

[ref225] Quinn D. M. (2017). Identity concealment: multilevel predictors, moderators, and consequences. J. Soc. Issues 73, 230–239. 10.1111/josi.12213

[ref226] Quinn D. M. (2018). “When stigma is concealable: the costs and benefits for health” in The Oxford handbook of stigma, discrimination, and health. eds. Major B. Dovidio J. F. Link B. G. (New York, NY: Oxford University Press), 287–299.

[ref227] Rajacich D. Kane D. Williston C. Cameron S. (2013). If they do call you a nurse, it is always a “male nurse”: experiences of men in the nursing profession. Nurs. Forum 48, 71–80. 10.1111/nuf.12008, PMID: 23379398

[ref228] Rasinski H. Czopp A. (2010). The effect of target status on witnesses’ reactions to confrontations of bias. Basic Appl. Soc. Psychol. 32, 8–16. 10.1080/01973530903539754

[ref229] Rasinski H. M. Geers A. L. Czopp A. M. (2013). “I guess what he said wasn’t that bad”: dissonance in nonconfronting targets of prejudice. Personal. Soc. Psychol. Bull. 39, 856–869. 10.1177/0146167213484769, PMID: 23813423

[ref230] Remedios J. D. Snyder S. H. (2018). Intersectional oppression: multiple stigmatized identities and perceptions of invisibility, discrimination, and stereotyping. J. Soc. Issues 74, 265–281. 10.1111/josi.12268

[ref231] Richman L. S. VanDellen M. Wood W. (2011). How women cope: being a numerical minority in a male-dominated profession. J. Soc. Issues 67, 492–509. 10.1111/j.1540-4560.2011.01711.x

[ref232] Riordan C. M. Schaffer B. S. Stewart M. M. (2004). “Relational demography within groups: through the lens of discrimination” in Discrimination at work: The psychological and organizational bases. eds. Dipboye R. L. Collella A. (Mahwah, NJ: Erlbaum), 37–62.

[ref233] Roberson Q. M. Roberson L. Kulik C. T. Tan R. Y. (2013). “Effective diversity training” in The Oxford handbook of diversity and work. ed. Roberson Q. (New York, NY, US: Oxford University Press).

[ref234] Rosenthal L. Levy S. R. London B. Lobel M. Bazile C. (2013). In pursuit of the MD: the impact of role models, identity compatibility, and belonging among undergraduate women. Sex Roles 68, 464–473. 10.1007/s11199-012-0257-9, PMID: 24497671 PMC3909517

[ref235] Rudman L. A. Glick P. (2001). Prescriptive gender stereotypes and backlash toward agentic women. J. Soc. Issues 57, 743–762. 10.1111/0022-4537.00239,

[ref236] Rudman L. A. Mescher K. (2013). Penalizing men who request a family leave: is flexibility stigma a femininity stigma? J. Soc. Issues 69, 322–340. 10.1111/josi.12017

[ref237] Ryan M. K. Haslam S. A. Hersby M. D. Kulich C. Atkins C. (2008). Opting out or pushed off the edge? The glass cliff and the precariousness of women’s leadership positions. Soc. Personal. Psychol. Compass 2, 266–279. 10.1111/j.1751-9004.2007.00007.x

[ref238] Saguy T. Dovidio J. F. (2013). Insecure status relations shape preferences for the content of intergroup contact. Personal. Soc. Psychol. Bull. 39, 1030–1042. 10.1177/0146167213487078, PMID: 23719622

[ref239] Saguy T. Dovidio J. F. Pratto F. (2008). Beyond contact: intergroup contact in the context of power relations. Personal. Soc. Psychol. Bull. 34, 432–445. 10.1177/0146167207311200, PMID: 18272809

[ref240] Saguy T. Kteily N. (2014). Power, negotiations, and the anticipation of intergroup encounters. Eur. Rev. Soc. Psychol. 25, 107–141. 10.1080/10463283.2014.957579

[ref241] Schmader T. Beilock S. (2012). “An integration of processes that underlie stereotype threat” in Stereotype threat: Theory, process, and application. eds. Inzlicht M. Schmader T. (New York, NY: Oxford University Press), 34–50.

[ref242] Schmader T. Johns M. (2003). Converging evidence that stereotype threat reduces working memory capacity. J. Pers. Soc. Psychol. 85, 440–452. 10.1037/0022-3514.85.3.440 14498781

[ref243] Schmader T. Johns M. Forbes C. (2008). An integrated process model of stereotype threat effects on performance. Psychol. Rev. 115, 336–356. 10.1037/0033-295X.115.2.336, PMID: 18426293 PMC2570773

[ref244] Schmader T. Sedikides C. (2018). State authenticity as fit to environment: the implications for social identity for fit, authenticity and self-segregation. Personal. Soc. Psychol. Rev. 22, 228–259. 10.1177/1088868317734080 28975851

[ref245] Schmitt M. T. Branscombe N. R. Postmes T. Garcia A. (2014). The consequences of perceived discrimination for psychological well-being: a meta-analytic review. Psychol. Bull. 140, 921–948. 10.1037/a0035754, PMID: 24547896

[ref246] Seibt B. Förster J. (2004). Stereotype threat and performance: how self-stereotypes influence processing by inducing regulatory foci. J. Pers. Soc. Psychol. 87, 38–56. 10.1037/0022-3514.87.1.38, PMID: 15250791

[ref247] Sekaquaptewa D. Thompson M. (2002). The differential effects of solo status on members of high- and low-status groups. Personal. Soc. Psychol. Bull. 28, 694–707. 10.1177/0146167202288013

[ref248] Sekaquaptewa D. Thompson M. (2003). Solo status, stereotype threat, and performance expectancies: their effects on women’s performance. J. Exp. Soc. Psychol. 39, 68–74. 10.1016/S0022-1031(02)00508-5

[ref249] Seligman M. E. P. (1971). Phobias and preparedness. Behav. Ther. 2, 307–320. 10.1016/S0005-7894(71)80064-3 27816071

[ref250] Shelton J. N. Alegre J. M. Son D. (2010). Social stigma and disadvantage: current themes and future prospects. J. Soc. Issues 66, 618–633. 10.1111/j.1540-4560.2010.01666.x

[ref251] Shelton J. N. Richeson J. A. Salvatore J. (2005). Expecting to be the target of prejudice: implications for interethnic interactions. Personal. Soc. Psychol. Bull. 31, 1189–1202. 10.1177/0146167205274894 16055639

[ref252] Shelton J. N. Richeson J. A. Salvatore J. Hill D. M. (2006). “Silence is not golden: the intrapersonal consequences of not confronting prejudice” in Stigma and group inequality: Social psychological approaches. eds. Levin S. Van Laar C. (Mahwah, NJ: Erlbaum), 65–81.

[ref253] Sherman D. K. Cohen G. L. (2002). Accepting threatening information: self-affirmation and the reduction of defensive biases. Curr. Dir. Psychol. Sci. 11, 119–123. 10.1111/1467-8721.00182

[ref254] Sherman D. K. Kinias Z. Major B. Kim H. S. Prenovost M. (2007). The group as a resource: reducing biased attributions for group success and failure via group affirmation. Personal. Soc. Psychol. Bull. 33, 1100–1112. 10.1177/0146167207303027, PMID: 17630262

[ref255] Shnabel N. Nadler A. Ullrich J. Dovidio J. F. Carmi D. (2009). Promoting reconciliation through the satisfaction of the emotional needs of victimized and perpetrating group members: the needs-based model of reconciliation. Personal. Soc. Psychol. Bull. 35, 1021–1030. 10.1177/0146167209336610, PMID: 19498070

[ref256] Simon S. O’Brien L. T. (2015). Confronting sexism: exploring the effect of nonsexist credentials on the costs of target confrontations. Sex Roles 73, 245–257. 10.1007/s11199-015-0513-x

[ref257] Smith J. L. Sansone C. White P. H. (2007). The stereotyped task engagement process: the role of interest and achievement motivation. J. Educ. Psychol. 99, 99–114. 10.1037/0022-0663.99.1.99

[ref258] Somvadee C. Morash M. (2008). Dynamics of sexual harassment for policewomen working alongside men. Policing 31, 485–498. 10.1108/13639510810895821

[ref259] Spears R. Doosje B. Ellemers N. (1997). Self-stereotyping in the face of threats to group status and distinctiveness: the role of group identification. Personal. Soc. Psychol. Bull. 23, 538–553. 10.1177/0146167297235009

[ref260] Spencer S. J. Logel C. Davies P. G. (2016). Stereotype threat. Annu. Rev. Psychol. 67, 415–437. 10.1146/annurev-psych-073115-103235, PMID: 26361054

[ref261] Spencer-Rodgers J. Major B. Foster D. E. Peng K. (2016). The power of affirming group values: group affirmation buffers the self-esteem of women exposed to blatant sexism. Self Identity 15, 413–431. 10.1080/15298868.2016.1145593, PMID: 27867318 PMC5114007

[ref262] Ståhl T. Van Laar C. Ellemers N. (2012a). The role of prevention focus under stereotype threat: initial cognitive mobilization is followed by depletion. J. Pers. Soc. Psychol. 102, 1239–1251. 10.1037/a0027678 22409487

[ref263] Ståhl T. Van Laar C. Ellemers N. Derks B. (2012b). Searching for acceptance: prejudice expectations direct attention towards social acceptance cues when under a promotion focus. Group Process. Intergroup Relat. 15, 523–538. 10.1177/1368430211435485

[ref264] Steele C. M. Aronson J. (1995). Stereotype threat and the intellectual test performance of African Americans. J. Pers. Soc. Psychol. 69, 797–811. 10.1037/0022-3514.69.5.797, PMID: 7473032

[ref265] Steele C. M. Spencer S. J. Aronson J. (2002). “Contending with group image: the psychology of stereotype and social identity threat” in Advances in experimental social psychology. ed. Zanna M. P. (San Diego, CA: Academic Press), 379–440.

[ref266] Stephens N. M. Fryberg S. A. Markus H. R. Johnson C. S. Covarrubias R. (2012a). Unseen disadvantage: how American universities’ focus on independence undermines the academic performance of first-generation college students. J. Pers. Soc. Psychol. 102, 1178–1197. 10.1037/a0027143 22390227

[ref267] Stephens N. M. Levine C. S. (2011). Opting out or denying discrimination? How the framework of free choice in American society influences perceptions of gender inequality. Psychol. Sci. 22, 1231–1236. 10.1177/0956797611417260 21934136

[ref268] Stephens N. M. Markus H. R. Phillips L. T. (2014). Social class culture cycles: how three gateway contexts shape selves and fuel inequality. Annu. Rev. Psychol. 65, 611–634. 10.1146/annurev-psych-010213-115143, PMID: 24079532

[ref269] Stephens N. M. Townsend S. S. M. Hamedani M. G. Destin M. Manzo V. (2015). A difference-education intervention equips first-generation college students to thrive in the face of stressful college situations. Psychol. Sci. 26, 1556–1566. 10.1177/0956797615593501 26290523

[ref270] Stephens N. M. Townsend S. S. M. Markus H. R. Phillips L. T. (2012b). A cultural mismatch: independent cultural norms produce greater increases in cortisol and more negative emotions among first-generation college students. J. Exp. Soc. Psychol. 48, 1389–1393. 10.1016/j.jesp.2012.07.008

[ref271] Sterk N. Meeussen L. Van Laar C. (2018). Perpetuating inequality: junior women do not see queen bee behavior as negative but are nonetheless negatively affected by it. Front. Psychol. 9:1690. 10.3389/fpsyg.2018.01690, 30294289 PMC6159757

[ref272] Stevens F. G. Plaut V. C. Sanchez-Burks J. (2008). Unlocking the benefits of diversity: all-inclusive multiculturalism and positive organizational change. J. Appl. Behav. Sci. 44, 116–133. 10.1177/0021886308314460

[ref273] Sutton R. M. Elder T. J. Douglas K. M. (2006). Reactions to internal and external criticism of outgroups: social convention in the intergroup sensitivity effect. Personal. Soc. Psychol. Bull. 32, 563–575. 10.1177/0146167205282992 16702151

[ref274] Swim J. K. Cohen L. L. Hyers L. L. (1998). “Experiencing everyday prejudice and discrimination” in Prejudice: The target's perspective. eds. Swim J. K. Stangor C. (San Diego, CA, US: Academic Press), 37–60.

[ref275] Swim J. K. Hyers L. L. (1999). Excuse me–What did you just say?!: women’s public and private responses to sexist remarks. J. Exp. Soc. Psychol. 35, 68–88. 10.1006/jesp.1998.1370

[ref276] Swim J. K. Stangor C. (1998). Prejudice: The target’s perspective. San Diego, CA: Academic Press.

[ref277] Swim J. K. Thomas M. A. (2006). “Responding to everyday discrimination: a synthesis of research on goal directed, self-regulatory coping behaviors” in Stigma and group inequality: Social psychological perspectives. eds. Levin S. Van Laar C. (Mahwah: Lawrence Erlbaum Associates Publishers), 105–126.

[ref278] Tajfel H. Turner J. C. (1979). “An integrative theory of intergroup conflict” in The social psychology of intergroup relations. eds. Austin W. G. Worchel S. (Monterey, CA: Brooks/Cole), 33–47.

[ref279] Turner J. C. (1981). Towards a cognitive redefinition of the social group. Curr. Psychol. Cogn. 1, 93–118.

[ref280] Turner J. C. Hogg M. A. Oakes P. J. Reicher S. D. Wetherell M. S. (1987). Rediscovering the social group: A self-categorization theory. Oxford, UK: Blackwell.

[ref281] Unzueta M. M. Binning K. R. (2012). Diversity is in the eye of the beholder: how concern for the in-group affects perceptions of racial diversity. Personal. Soc. Psychol. Bull. 38, 26–38. 10.1177/0146167211418528 21868494

[ref282] Van Breen J. A. Spears R. Kuppens T. De Lemus S. (2018). Subliminal gender stereotypes: who can resist? Personal. Soc. Psychol. Bull. 44, 1648–1663. 10.1177/0146167218771895, PMID: 29781373

[ref283] Van Dijk H. van Engen M. L. (2019). The flywheel effect of gender role expectations in diverse work groups. Front. Psychol. 10:976. 10.3389/fpsyg.2019.00976 31133926 PMC6513879

[ref284] Van Grootel S. Van Laar C. Meeussen L. Schmader T. Sczesny S. (2018). Uncovering pluralistic ignorance to change men’s communal self-descriptions, attitudes, and behavioral intentions. Front. Psychol. 9:1344. 10.3389/FPSYG.2018.01344 30147664 PMC6095955

[ref285] Van Knippenberg D. Van Ginkel W. P. Homan A. C. (2013). Diversity mindsets and the performance of diverse teams. Organ. Behav. Hum. Decis. Process. 121, 183–193. 10.1016/j.obhdp.2013.03.003

[ref286] Van Laar C. Bleeker D. Ellemers N. Meijer E. (2014). Ingroup and outgroup support for upward mobility: divergent responses to ingroup identification in low status groups. Eur. J. Soc. Psychol. 44, 563–577. 10.1002/ejsp.2046

[ref287] Van Laar C. Derks B. Ellemers N. (2013). Motivation for education and work in young Muslim women: the importance of value for ingroup domains. Basic Appl. Soc. Psychol. 35, 64–74. 10.1080/01973533.2012.746609

[ref288] Van Laar C. Derks B. Ellemers N. Bleeker D. (2010). Valuing social identity: consequences for motivation and performance in low-status groups. J. Soc. Issues 66, 602–617. 10.1111/j.1540-4560.2010.01665.x

[ref289] Van Laer K. (2018). The role of co-workers in the production of (homo)sexuality at work: a Foucauldian approach to the sexual identity processes of gay and lesbian employees. Hum. Relat. 71, 229–255. 10.1177/0018726717711236

[ref290] van Peer J. M. Roelofs K. Rotteveel M. van Dijk J. G. Spinhoven P. Ridderinkhof K. R. (2007). The effects of cortisol administration on approach–avoidance behavior: an event-related potential study. Biol. Psychol. 76, 135–146. 10.1016/j.biopsycho.2007.07.003 17728047

[ref291] Van Prooijen A. M. Ellemers N. (2015). Does it pay to be moral? How indicators of morality and competence enhance organizational and work team attractiveness. Br. J. Manag. 26, 225–236. 10.1111/1467-8551.12055

[ref292] Veldman J. Meeussen L. Van Laar C. (2019). A social identity perspective on the social-class achievement gap: academic and social adjustment in the transition to university. Group Process. Intergroup Relat. 22, 403–418. 10.1177/1368430218813442

[ref293] Veldman J. Meeussen L. Van Laar C. Phalet K. (2017). Women (do not) belong here: gender-work identity conflict among female police officers. Front. Psychol. 8:130. 10.3389/fpsyg.2017.00130, PMID: 28220097 PMC5292822

[ref294] Vignoles V. L. (2011). “Identity motives” in Handbook of identity theory and research. eds. Schwartz S. J. Luyckx K. Vignoles V. L. (New York, NY, US: Springer Science + Business Media), 403–432.

[ref295] Vignoles V. L. Regalia C. Manzi C. Golledge J. Scabini E. (2006). Beyond self-esteem: influence of multiple motives on identity construction. J. Pers. Soc. Psychol. 90, 308–333. 10.1037/0022-3514.90.2.308, PMID: 16536653

[ref296] Von Hippel C. Issa M. Ma R. Stokes A. (2011). Stereotype threat: antecedents and consequences for working women. Eur. J. Soc. Psychol. 41, 151–161. 10.1002/ejsp.749

[ref297] von Hippel C. Kalokerinos E. K. Henry J. D. (2013). Stereotype threat among older employees: relationship with job attitudes and turnover intentions. Psychol. Aging 28, 17–27. 10.1037/a0029825, PMID: 22924658

[ref298] Walton G. M. Cohen G. L. (2007). A question of belonging: race, social fit, and achievement. J. Pers. Soc. Psychol. 92, 82–96. 10.1037/0022-3514.92.1.82, PMID: 17201544

[ref299] Wayne J. Cordeiro H. (2003). Who Is a good organizational citizen? Social perception of male and female employees who use family leave. Sex Roles 49, 233–246. 10.1023/A:1024600323316

[ref300] Weiss D. Lang F. (2012). “They” are old but “I” feel younger: age-group dissociation as a self-protective strategy in old age. Psychol. Aging 27, 153–163. 10.1037/a0024887, PMID: 21988154

[ref301] Wilder D. A. (1984). Predictions of belief of homogeneity and similarity following social categorization. Br. J. Soc. Psychol. 23, 323–333. 10.1111/j.2044-8309.1984.tb00648.x

[ref302] Williams D. R. Neighbors H. W. Jackson J. S. (2003). Racial/ethnic discrimination and health: findings from community studies. Am. J. Public Health 93, 200–208. 10.2105/AJPH.93.2.200, PMID: 12554570 PMC1447717

[ref303] Wills T. A. (1985). “Supportive functions of interpersonal relationships” in Social support and health. eds. Cohen S. Syme S. L. (San Diego, CA: Academic Press), 61–82.

[ref304] Wilson-Kovacs D. Ryan M. K. Haslam S. A. Rabinovich A. (2008). “Just because you can get a wheelchair in the building doesn’t necessarily mean that you can still participate”: barriers to the career advancement of disabled professionals. Disabil. Soc. 23, 705–717. 10.1080/09687590802469198

[ref305] Wright S. C. Taylor D. M. (1999). Success under tokenism: co-option of the newcomer and the prevention of collective protest. Br. J. Soc. Psychol. 38, 369–396. 10.1348/014466699164220

[ref306] Wright S. C. Taylor D. M. Moghaddam F. M. (1990). Responding to membership in a disadvantaged group: from acceptance to collective protest. J. Pers. Soc. Psychol. 58, 994–1003. 10.1037/0022-3514.58.6.994

[ref307] Wrosch C. Scheier M. F. Carver C. S. Schulz R. (2003). The importance of goal disengagement in adaptive self-regulation: when giving up is beneficial. Self Identity 2, 1–20. 10.1080/15298860309021

